# Antibacterial efficacy of *Solanum muricatum* aiton metabolites against methicillin-resistant *staphylococcus aureus*: Insights into bioactive compounds and molecular mechanisms

**DOI:** 10.1371/journal.pone.0338733

**Published:** 2025-12-26

**Authors:** Sarah Samir, Fathy A. Behery, Mohamed A. Zarka, Ruqaiah I. Bedaiwi, Hesham A. Abou-Zied, Usama Ramadan Abdelmohsen, Rehab Mahmoud Abd El-Baky, Mohamed A. Mawhoup, Mai Mahrous, Reem E. S. Abdelnaem, Gerhard Bringmann, Abeer H. Elmaidomy

**Affiliations:** 1 Departement of Pharmacognosy, Faculty of Pharmacy, Misr University for Science and Technology (MUST), 6th October city, Giza, Egypt; 2 Department of Pharmacognosy, Faculty of Pharmacy, Mansoura University, Mansoura, Egypt; 3 Department of Pharmacy, College of Pharmacy, Nursing and Medical Sciences, Riyadh Elm University, Riyadh, Saudi Arabia; 4 Department of Pharmacognosy, College of Pharmacy, The Islamic University, Najaf, Iraq; 5 Department of Medical Laboratory Technology, Faculty of Applied Medical Sciences, University of Tabuk, Tabuk, Saudi Arabia; 6 Department of Medicinal Chemistry, Faculty of Pharmacy, Deraya University, Minia, Egypt; 7 Deraya Center for Scientific Research, Deraya University, Minia, Egypt; 8 Department of Pharmacognosy, Faculty of Pharmacy, Minia University, Minia, Egypt; 9 Department of Microbiology and Immunology, Faculty of Pharmacy, Minia University, Minia, Egypt; 10 Department of Microbiology and Immunology, Faculty of Pharmacy, Deraya University, Minia, Egypt; 11 Institute of Organic Chemistry, University of Würzburg, Würzburg, Germany; 12 Department of Pharmacognosy, Faculty of Pharmacy, Beni-Suef University, Beni-Suef, Egypt; University of Buea, CAMEROON

## Abstract

The incidence of methicillin-resistant *Staphylococcus aureus* (MRSA) has been steadily increasing in Ethiopia over the past few decades. As a result, the need for new antibiotic classes has become imperative to combat the growing threat of multidrug-resistant bacteria, including MRSA. Phytochemical investigation of the aerial parts extract of the edible plant *Solanum muricatum* Aiton (F. Solanaceae) afforded eight known metabolites: kaempferol 3-*O*-gentiobioside (**1**), kaempferol 3-*O*-sambubioside (**2**), quercetin 3-*O*-rhamnoside (**3**), procyanidin A2 (**4**), procyanidin A2 3-*O*-glucoside (**5**), (2*S*)-2-hydroxy-3-[(9*Z*,12*Z*)-1-oxo-9,12-octadecadien-1-yl]oxy]propyl-*O-β*-D-galactopyranoside (**6**), palmitic acid (**7**), and linoleic acid (**8**). The structures of the isolated compounds were assigned by 1D and 2D NMR. The crude extract exhibited moderate anti-*Staphylococcus* activity (MIC = 196.8 µg/mL), while compound **1** (kaempferol 3-*O*-gentiobioside) showed the strongest inhibitory effect (MIC = 8.3 µM), followed by compounds **2** and **3** (MIC = 10.2 and 11.2 µM, respectively). These compounds significantly reduced MRSA biofilm formation by up to 75.09% at sub-MIC concentrations (*p* < 0.05). Checkerboard assays revealed synergistic interactions among compounds **1**, **2**, and **3** and between these compounds and gentamicin (FICI < 0.5), suggesting enhanced therapeutic potential when combined. An integrated computational approach combining protein-protein interaction (PPI) network analysis, molecular docking, and molecular dynamics (MD) simulations was employed. The PPI network analysis, constructed using the STRING and STITCH databases, revealed critical MRSA-associated targets and their interactions with bioactive compounds from *S. muricatum*. Network hub analysis identified key immune-regulatory and antibacterial resistance-related proteins, suggesting potential intervention points. Molecular docking results identified kaempferol 3-gentiobioside (compound **1**) as the most potent inhibitor of APH(3’)-IIIa, with strong binding energy and interactions with key catalytic residues. Further 150 ns MD simulations confirmed the stability of the compound **1**-APH(3’)-IIIa complex, as evidenced by minimal RMSD fluctuations, sustained hydrogen bonding, stable protein compactness (Rg), and favorable potential energy values.

## 1. Introduction

*Staphylococcus aureus* is a common Gram-positive bacterium that can colonize the nasal passages as well as the skin, axillae, perineum, and pharynx [1]. It can cause a broad spectrum of infectious diseases, from minor skin and soft tissue infections to severe conditions such as endocarditis, osteomyelitis, pneumonia, and bacteremia. The global overuse of antibiotics has led to the rise of antibiotic-resistant strains. Methicillin-resistant *S. aureus* (MRSA) arises from the acquisition of the *staphylococcal* cassette chromosome mec (SCCmec), which includes the mecA gene responsible for producing an altered penicillin-binding protein 2a (PBP2a) with reduced affinity for all *β*-lactam antibiotics. The significant prevalence of both hospital-acquired MRSA (HA-MRSA) and community-acquired MRSA (CA-MRSA) is a major global concern. HA-MRSA is associated with higher mortality rates, prolonged hospitalization, and increased healthcare costs [2].

Vancomycin can be considered as the last line of defense for severe MRSA infections [3]. However, it is associated with side effects such as nephrotoxicity, hypotension, and hypersensitivity reactions, necessitating drug monitoring [4]. Consequently, there is an urgent need to identify new alternative anti-MRSA agents with lower toxicity, and one promising approach is the use of plant-derived compounds.

The genus *Solanum* is considered to be one of the largest and most complex genera among the Angiosperms, and the most representative and largest genus of the family Solanaceae [5]. It is comprised of about 2000 species distributed across subtropical and tropical regions. The genus *Solanum* is rich in economically significant species; the food crops include *S. aethiopicum*, *S. anguivi, S. lycopersicum, S. melongena, S. muricatum, S. torvum,* and *S. tuberosum* [5]. Ornamental species include *S. aviculare, S. capsicastrum, S. crispum, S. laciniatum, S. laxum, S. pseudocapsicum, S. rantonnetii, S. seaforthianum,* and *S. wendlandii* [5]. The medicinal species of *Solanum* were reported to contain steroidal saponins, steroidal alkaloids, pregnane glycosides, terpenes, flavonoids, lignans, other types of alkaloids, sterols, phenolic compounds, coumarins and coumestans, coumarinolignoids, fatty acids and esters [5]. A series of pharmacological studies have been carried out to verify and validate the traditional medicinal applications of many plants in this genus. The studied pharmacological activities include analgesic, anthelminthic, antiallergic, anti-anemic, anti-asthmatic, antibacterial, anti- cancer, anti-convulsant, anti-depressant, anti-diabetic, anti-fungal, antihistaminic, antihypertensive, anti-inflammatory, antileishmanial, antimelanogenetic, anti-molluscicidal, antinociceptive, anti-psoriatic, antiplasmodial, antiprotozoal, anti-trypanosomal, antiurolithiatic, antiviral, cardio-vascular, diuretic, hepatoprotective, hypolipidemic, mosquito larvicidal, nephrotoxic, spasmolytic, schistosomicidal, and vasorelaxant activities [5].

The pepino (*Solanum muricatum* Aiton) is a well-known cultigen of the Andes, with Southern Colombia/Northern Ecuador as the main center of diversity [6]. Pepinos are described by early Spanish chroniclers as being cultivated on the coast. Pepino has been known by its different common names viz. cachuma, melonpaere, pepino, appelmeloen, meloen peer, and peermeloen [6]. The pepino fruit resembles a melon (*Cucumis melo*) in color and its flavor recalls a succulent mixture of honeydew and cucumber, thus it is also sometimes called pepino melon or melon pear, but pepinos are only very distantly related to melons and pears. It is a ground cover and trailing plant. This species is, however, a close relative of other nightshades cultivated for their fruit, including the tomato and the eggplant, and the pepino fruit closely resembles that of these relatives [6]. Fruits and leaves of pepino were found to contain alkaloids, flavonoids, and tannins through phytochemical screening studies [7].

In the present study, eight compounds were isolated and identified from the aerial parts methanolic extract of *S. muricatum*. The isolated compounds were evaluated for their anti-MRSA activities. Additionally, the structures of the isolated compounds underwent molecular docking to determine the possible mechanisms responsible for their activities.

## 2. Materials and methods

### 2.1. Plant material

*S. muricatum* aerial parts (leaves, flowers, and stems) were collected in August 2024 from the Faculty of Pharmacy, Beni-Suef. The material was taxonomically identified by Dr. Abd ElHalim A. Mohammed (Department of Flora and Phytotaxonomy Research, Dokki, Cairo, Egypt). A voucher specimen (2023-BuPD 105) is kept at the Department of Pharmacognosy, Faculty of Pharmacy, Beni-Suef University, Egypt.

### 2.2. Chemicals and reagents

The solvents used in this work were dichloromethane (DCM), and methanol (MeOH), purchased from El-Nasr Company for Pharmaceuticals and Chemicals (Egypt). Deuterated solvents used for spectroscopic analyses were purchased from Sigma-Aldrich (Saint Louis, Missouri, USA), including CD_3_OD. Column chromatography (CC) was performed using silica gel 60 (63–200 μm, E. Merck, Sigma-Aldrich), and Sephadex LH-20 (0.25–0.1 mm, GE Healthcare, Sigma-Aldrich). Thin-layer chromatography (TLC) was carried out on pre-coated silica gel 60 GF_254_ plates (E. Merck, Darmstadt, Germany; 20 × 20 cm, 0.25 mm in thickness). Spots were visualized by spraying with *P-*anisaldehyde (PAA) reagent (85: 5 : 10 : 0.5, with absolute EtOH : sulfuric acid : G.A.A. : *para*-anisaldehyde), followed by heating to 110 °C [8].

### 2.3. Spectral analysis

Proton ^1^H and Distortionless Enhancement by Polarization Transfer-Q (DEPT-Q) NMR spectra were recorded at 400 and 100 MHz, respectively. Tetramethylsilane (TMS) was used as an internal standard in CD_3_OD, using the residual solvent peak (*δ*_H_ = 3.34 and 4.78; and *δ*_C_ = 49.9) as the reference. Measurements were performed on a Bruker Advance III 400 MHz with a BBFO Smart Probe and a Bruker 400 MHz EON Nitrogen-Free Magnet (Bruker AG, Billerica, MA, USA). Carbon multiplicities were determined using a DEPT-Q experiment.

### 2.4. Extraction and fractionation of Solanum muricatum

*S. muricatum* aerial parts (1 kg) were collected, air-dried in the shade for one weak, then finely powdered using an OC-60B/60B grinding machine (60–120 mesh, Henan, Mainland China). The powder was extracted by maceration using 70% methanol (5 L, 3 × , 7 d each) at room temperature, and concentrated under vacuum at 45 °C using a rotary evaporator (Buchi Rotavapor R-300, Cole-Parmer, Vernon Hills, IL, USA) to afford 100 g crude extract then kept at 4 °C for biological and phytochemical investigations [9–17].

### 2.5. Isolation and purification of Solanum muricatum compounds

**Fig 1:**
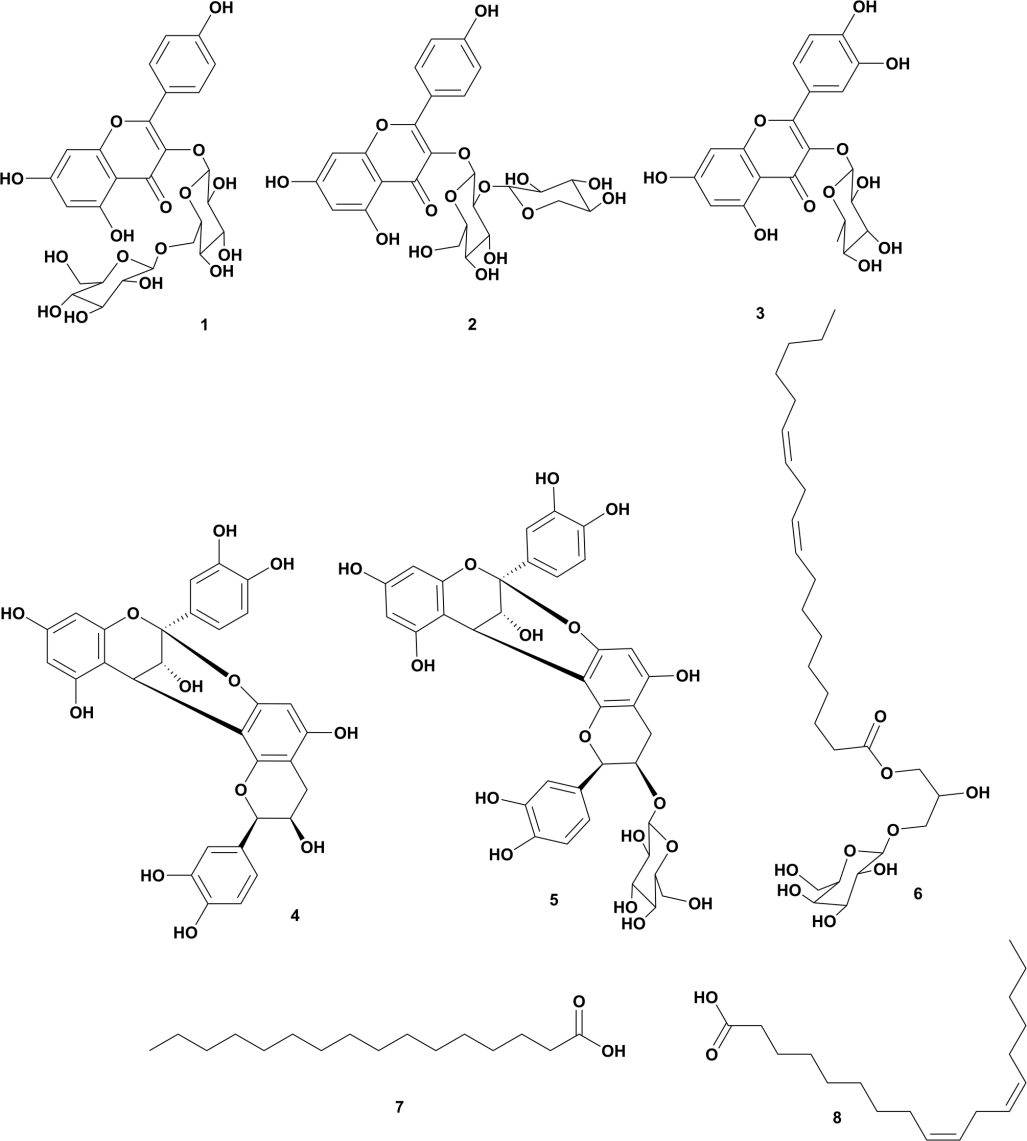
Structures of compounds isolated from *Solanum muricatum.* Compounds identified as: kaempferol 3-*O*-gentiobioside (**1**), kaempferol 3-*O*-sambubioside (**2**), quercetin 3-*O*-rhamnoside (**3**), procyanidin A2 (**4**), procyanidin A2 3-*O*-glucoside (**5**), (2S)-2-hydroxy-3-[(9Z,12Z)-1-oxo-9,12-octadecadien-1-yl]oxypropyl-O-β-D-galactopyranoside (**6**), palmitic acid (**7**), and linoleic acid (**8**).

The dried extract (50 g) was fractionated on polyamide using gradient elution starting with H_2_O and ending with MeOH. This resulted in six fractions, F1–F6, which were then chromatographed separately on Sephadex LH-20, eluted with MeOH. This was followed by chromatography on Si gel 60, eluting CH_2_Cl_2_-MeOH (gradient elution) to afford compounds **1** (kaempferol 3-*O*-gentiobioside, 15 mg), **2** (kaempferol 3-*O*-sambubioside, 34 mg), **3** (quercetin 3-*O*-rhamnoside, 70 mg), **4** (procyanidin A2, 10 mg), **5** (procyanidin A2 3-*O*-glucoside, 30 mg), **6** ((2S)-2-hydroxy-3-[(9Z,12Z)-1-oxo-9,12-octadecadien-1-yl]oxypropyl-O-β-D-galactopyranoside, 20 mg), **7** (palmitic acid, 35 mg), and **8** (linoleic acid, 14 mg), for the structures of compounds **1**–**8**, see Fig 1) [12–14,16–20].

### 2.6. Bacterial strains and growth condition

The methicillin-resistant *Staphylococcus aureus* (ATCC 43300) strain was stored at –80 °C in 2.5 M glycerol until use. For experimental assays, the strain was routinely cultured on Mueller-Hinton agar and grown in Mueller-Hinton broth at 37 °C for 18–24 h under aerobic conditions before each experiment [21, 22].

### 2.7. Screening for anti-Staphylococcus activity

The antibacterial activity assay was done using the method described by [23]. The plates were prepared by pouring nutrient agar media into sterile petri plates and allowed to set. The micro-organism was inoculated on the plates using a swab stick. A 4-mm cork borer was used to bore holes on the medium, and the bottom of each hole was sealed with a drop of molten agar to avoid seepage of the extract. Four holes were made on each petri plate, adequately spaced out. About 0.2 μL of the different concentrations of each tested compound, crude extract and gentamicin were introduced into the well. The petri plates were incubated at 37 °C for 24 h, after which the inhibition zones were measured using a ruler.

### 2.8. Minimum inhibitory concentration determination and testing the synergistic activity of the most active tested compounds

The minimum inhibitory concentrations (MIC) of the crude extract and of the tested compounds, dissolved in DMSO, were determined against methicillin-resistant *Staphylococcus aureus* (clinical isolate) using the micro broth dilution method according to the Clinical Laboratory and Standards Institute Guidelines [24]. The tested compounds were serially 2-fold diluted, and gentamicin was used as a reference. Briefly, an overnight culture of MRSA was used and standardized to have turbidity equivalent to 0.5 McFarland with Muller-Hinton broth. Equal volumes (100 µL) of serially diluted concentrations of the tested compounds were added to the wells of a 96-microtiter plate, followed by adding 10 µL of the microbial suspension. The plates were incubated for 24 h at 37 °C. The test was conducted in triplicate. MIC was determined to have the lowest concentration of the tested compounds that inhibit visible growth.

To determine the Minimum bactericidal concentration, 10 µL from each well was placed on the surface of Muller-Hinton agar and incubated at 37 °C for 24 h. Plates with the lowest concentration show no growth, were considered as the MBC of the tested compounds.

### 2.9. Testing the synergistic activity of the most active tested compounds by the checkerboard assay method

Testing the synergistic activity between the active compounds with each other and with gentamicin by the checkerboard assay method [25]:

Stock solutions were prepared from each compound, then serial two-fold dilutions of each drug to at least double the MIC were used to determine the MICs of compounds **1**, **2**, and **3** with each other and with gentamicin. One hundred µL of TSB was distributed in each well of 96-well microtiter plates. Different dilutions of the tested compounds were added to the TSB prefilled wells. Each well was inoculated with 10 µL of MRSA suspension (equivalent to 0.5 McFarland). Plates were incubated at 37 °C for 24 h. MICs of the tested agents were determined.

The FICI values were then calculated using the following formula:


FICI=FIC (A)+FIC (B)

FIC (A or B) = MIC (A or B) in combination/MIC (A or B) alone.

The FICI values were interpreted as follows: < 0.5 = synergistic; 0.5 ≤ FICI ≤ 1.0 = additive; 1.0 < FICI ≤ 4.0–4.0 = indifferent (non-interactive); > 4.0 = antagonistic.

### 2.10. Effect of the crude extract and tested compounds on biofilm formation by methicillin-resistant Staphylococcus aureus (MRSA)

The effect of the crude extract, and tested compounds on the adherence of the tested strain and their ability to form biofilm was tested according to the method described by [26], with some modifications. The test was performed in 96-well polystyrene microtiter plates (Tarson, India). The microbial suspensions were prepared from the overnight-grown culture, and the turbidity of the suspension was adjusted to 0.7 OD_595_ (1 × 10^9^ CFU/mL). In each well, 100 µL of Tryptic Soy Broth (TSB, Difco Laboratories) broth supplemented with 0.5% glucose was placed in each well, sub-MIC concentrations of tested crude extract, and tested compounds were added, followed by the addition of 10 μL of the microbial suspension. Plates were incubated for 18 h at < 37 °C, washed with phosphate buffer saline (PBS) and biofilms were fixed and stained with Crystal Violet (Sigma-Aldrich Co.). Biofilm formation was quantified by the addition of 95% ethanol to the crystal violet-stained wells and recording the absorbance at 595 nm using a microplate reader (Multiskan spectrum; Finland). Gentamicin was used as a reference. The test was conducted in triplicate.


$$\text{Percentage}\;\text{of}\;\text{inhibition}\;(\%)=\text{100}\;–\;({\text{OD}}_{595}\;\text{of}\;\text{test}\;\text{wells}/{\text{OD}}_{595}\;\text{of}\;\text{control}\;\text{well})\times \text{100}$$

### 2.11. Protein-protein interaction network

To investigate the molecular interactions associated with the anti-*Staphylococcus* potential of *S. muricatum* aerial parts, key phytochemicals identified through experimental studies were analyzed using the STRING database, a well-established platform for constructing protein-protein interaction (PPI) networks. The objective was to understand how these bioactive compounds interact within the human host system, particularly in relation to their antibacterial effects against drug-resistant pathogens such as MRSA. A confidence score threshold of 0.4 was applied to ensure the inclusion of only highly relevant interactions in the analysis. Network visualization and interpretation were performed using Cytoscape, a robust tool for biological network analysis. This step was essential in identifying hub phytochemicals with significant connectivity, signifying their potential influence on critical biological pathways related to anti-*Staphylococcus* activity. The CytoHubba plugin in Cytoscape was employed for further refinement of the network, utilizing degree-based methods to highlight key bioactive components. Identifying these core phytochemicals provides deeper insights into their molecular interactions and highlights promising candidates for enhancing antibacterial efficacy. Recognizing these hub molecules is a crucial step toward developing novel therapeutic strategies, particularly in combating resistant bacterial strains like MRSA.

### 2.12. Gene ontology and Kyoto Encyclopedia of Genes and Genomes pathway enrichment

To gain deeper insights into the biological roles and molecular interactions of genes associated with MRSA, gene ontology (GO) and Kyoto encyclopedia of genes and genomes (KEGG) pathway enrichment analyses were conducted. These analyses provided insights from three perspectives: biological processes (BP), which describe the functional roles of genes in biological activities; cellular components (CC), which identify specific cellular locations where gene or protein activity occurs; and Molecular Functions (MF), which define the biochemical roles and interactions of the genes. The GO enrichment analysis was conducted using the STRING analyzer tool, with a strict false-discovery rate (FDR) cutoff of < 0.05 to ensure statistical significance. The results were visually represented. This systematic approach facilitated a deeper understanding of gene functions and their complex interactions in MRSA, identifying crucial targets for potential therapeutic interventions. The integration of advanced bioinformatics tools in this analysis enhances our ability to unravel the complex biological networks associated with MRSA, ultimately contributing to the development of more effective antibacterial treatments.

### 2.13. Molecular docking studies

Molecular docking was employed to validate the findings from network pharmacology by simulating the binding interactions between key proteins and potential drug candidates. The docking studies were conducted using the Discovery Studio Client platform, which facilitated the identification of promising compounds with high affinity for crucial MRSA-related targets. The crystal structure of the complex of butirosin A (which is an aminoglycoside antibiotic) with 3’,5“-aminoglycoside phosphotransferase type IIIa AMPPNP (PDB ID: 3TM0) was obtained from the RCSB Protein Data Bank (http://www.rcsb.org/) and prepared for molecular docking analysis. Protein preparation was carried out in UCSF Chimera, where non-essential molecular components, including water molecules, ions, and heteroatoms, were removed. All ligands were retrieved in 2D SDF format and converted to 3D structures. Ligands were protonated and assigned Gasteiger partial charges. The final structures were exported in mol2 format for docking. Butirosin A, the co-crystallized ligand in the APH(3’)-IIIa crystal structure (PDB ID: 3TM0), was used to define the active binding pocket and validate docking accuracy. Kanamycin A, a clinically relevant aminoglycoside antibiotic, was included as a reference control ligand for comparative analysis of binding affinity and interaction profile. The co-crystallized ligand, butirosin A, an aminoglycoside antibiotic, was analyzed to determine the active binding pocket and key interacting residues crucial for ligand recognition. The protein structure was carefully examined for any missing residues or atoms that could affect ligand binding and docking precision. To enhance docking accuracy, energy minimization was performed using the CHARMM force field. The docking simulations were executed using the CDOCKER algorithm, which generated manifold ligand conformations through random sampling and simulated annealing. A total of 40 docking runs per ligand were conducted to ensure comprehensive sampling of the binding pocket and potential interaction modes. Docking poses were ranked based on binding energy scores, with the top three conformations selected for further evaluation. The most favorable pose was identified based on the lowest binding energy, minimal RMSD, and strong interactions with critical active site residues identified from the kanamycin A binding pocket analysis. This systematic docking approach provided significant insights into the inhibitory potential of the selected compounds, reinforcing their relevance as drug candidates targeting MRSA-resistance mechanisms.

### 2.14. Molecular dynamics simulation (MDS)

To further validate the molecular docking results, molecular dynamics (MD) simulations were conducted using GROMACS 2023.5. The protein-ligand complexes were initially prepared in UCSF Chimera, where hydrogen atoms were added to ensure proper protonation states. To accurately model interactions, the CHARMM36 force field was applied for the protein, while CGenFF was used for ligand parameterization. The system was solvated in a triclinic water box employing the TIP3P water model, ensuring a minimum buffer distance of 1 nm around the protein-ligand complex. To maintain system neutrality, sodium chloride (NaCl) ions were added, with an overall salt concentration adjusted to 150 mM to mimic physiological conditions.

Energy minimization was carried out using the steepest-descent algorithm, followed by two equilibration phases: an NVT ensemble maintained at 300 K using the V-rescale thermostat with positional restraints on the protein and ligand, and an NPT ensemble maintained at 1.0 bar using the Berendsen barostat to ensure proper system stabilization. After equilibration, a 150-ns production MD simulation was performed with positional restraints removed, allowing the full flexibility of the molecular system. Trajectory snapshots were recorded every 10 ps to monitor root-mean-square deviation (RMSD) and binding energy fluctuations, assessing the structural stability and binding affinity of the proposed drug candidates. This rigorous MD approach provided dynamic insights into ligand stability and interaction strength, reinforcing the validity of the selected compounds against MRSA.

### 2.15. Statistical analysis

The data were tabulated using the statistical programmed GraphPad Prism version-9 (GraphPad, La-Jolla, CA, USA). All experiments were performed at least three times. *p < 0.05, Student’s t-test.

## 3. Results and discussion

### 3.1. Phytochemical investigation of Solanum muricatum

Based on_-_the physicochemical, chromatographic-properties, the-spectral-analyses-from UV, ^1^H, and-DEPT-Q-NMR, as well as-comparisons-with the-literature, the crude extract of *S. muricatum* aerial parts afforded eight known compounds, which were identified as kaempferol 3-*O*-gentiobioside (**1**) [27], kaempferol 3-*O*-sambubioside (**2**) [28], quercetin 3-*O*-rhamnoside (**3**) [29], procyanidin A2 (**4**) [30], procyanidin A2 3-*O*-glucoside (**5**) [30], (2*S*)-2-hydroxy-3-[(9*Z*,12*Z*)-1-oxo-9,12-octadecadien-1-yl]oxy]propyl-*O-β*-D-galactopyranoside (**6**) [31], palmitic acid (**7**) [32], and linoleic acid (**8**) [32], (S1-S4Tables in S1 File, Fig 1, S1-S14 Tables in S1 File).

### 3.2. Anti-Staphylococcus susceptibility testing

#### 3.2.1. Screening of the antibacterial activity of the tested compounds against MRSA:

**Fig 2:**
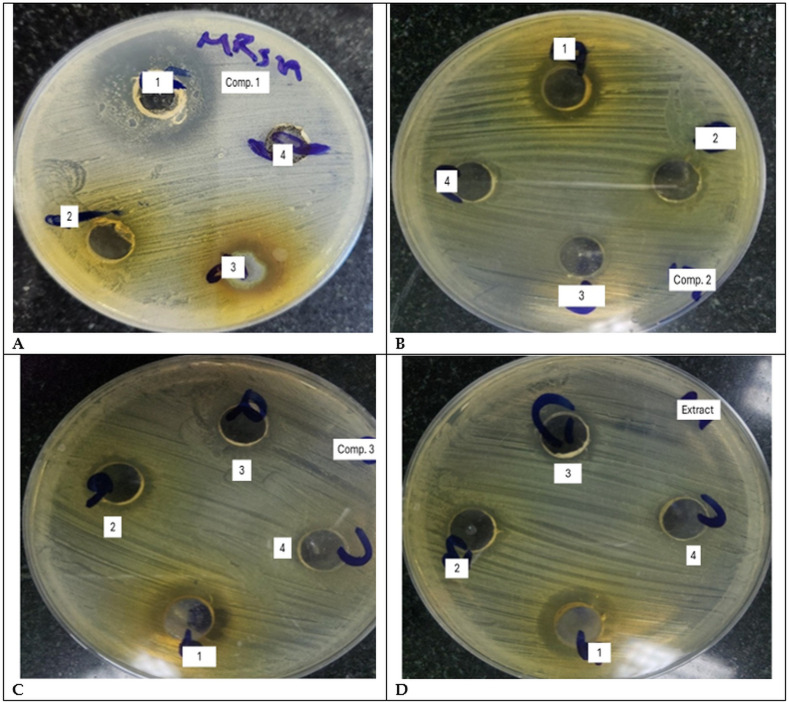
Antibacterial activity of the tested compounds 1, 2, and 3, and the extract against the tested *methicillin-resistant Staphylococcus aureus* (MRSA). The effect of 2-fold dilutions of the tested compounds, serially diluted from 1 (the highest concentration to 4 (the lowest concentration). **(A)**: compound **1**; **(B)**: compound **2**; **(C)**: compound **3**; and **(D)**: the extract.

Screening of the anti-*Staphylococcus* activity of eight compounds and the extract was performed using the well agar diffusion method, starting from 100 µM to 12.55 µM. Compound **1** showed the highest activity, represented by zone diameters ranging from 30 mm to 12 mm for the lower concentration. While the other tested compounds showed smaller zones of inhibition at the same concentration (Fig 2).

#### 3.2.2. *Determination of minimum inhibitory concentration of the crude extract, and of the tested compounds against MRSA:.*

In this investigation, the crude extract, and isolated compounds from *S. muricatum* aerial parts methanolic extract (compounds **1**–**8**), were evaluated against methicillin-resistant *Staphylococcus aureus* (ATCC 43300), using the broth dilution method. The results showed that compound **1** (MIC = 8.3 µM) was the most active, followed by compounds **2** and **3** (MIC = 10.2 µM and 11.2 µM, respectively) (Table 1). Gentamicin was used as a positive control (MIC = 4 µM) (Table 1).

**Table 1:** The anti-MRSA activity of *Solanum muricatum* aerial parts crude extract and isolated compounds, as determined using the broth dilution method.

Tested compound	MIC (µM)	MBC (µM)
1	8.3	16.5
2	10.2	20.4
3	11.2	22.4
4	8.85 × 10^3^	26.5 × 10^3^
5	4.29 × 10^3^	8.85 × 103
6	2.614 × 10^3^	5.25 × 10^3^
7	1.7 × 10^3^	1.7 × 10^3^
8	1.2 × 10^3^	2.4 × 10^3^
Extract	196.8 (µg/mL)	392 (µg/mL)
Gentamicin	4	8

The GraphPad Prism 9 software was used to calculate the minimum inhibitory concentration MIC (µM) values. All experiments were performed at least three times. *p < 0.05, Student’s *t*-test.

#### 3.2.3. *Testing the synergistic activity of the most active tested compounds by the checkerboard assay method:.*

Our results showed the synergistic potential of the combinations of the tested compounds **1** and **2**, **1** and **3**, while combination of compound **1** with compound **3** showed additive activity. On the other hand, compound **1**/gentamicin combination and compound **2**/gentamicin combination showed synergistic activity but combination of gentamicin with compound **3** showed additive activity (Table 2).

**Table 2:** Determination of the combination effect of the tested most active compounds with each other and with gentamicin.

Tested combination		FIC A	FIC B	FICI
A	B			
Comp. 1	Comp. 2	0.2	0.2	0.4
Comp. 1	Comp. **3**	0.117	0.234	0.351
Comp. 2	Comp. **3**	0.34	0.288	0.628
Gentamicin	Comp. **1**	0.125	0.16	0.285
Gentamicin	Comp. **2**	0.25	0.163	0.413
Gentamicin	Comp. **3**	0.25	0.284	0.53

FIC: Fractional inhibitory concentration; FICI: Fractional inhibitory concentration index.

### 3.3. *Effect of the crude extract and the tested compounds on biofilm formation by MRSA*

**Fig 3:**
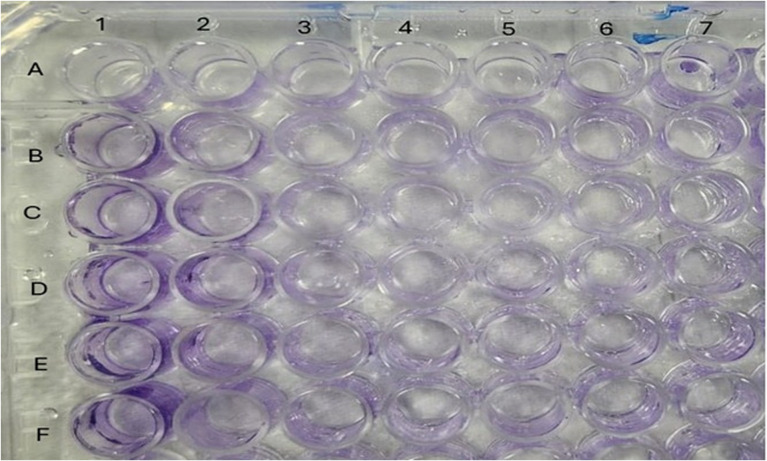
Effect of the tested compounds on biofilm formation by the tested isolate. **(A)**: negative control; **(B)**: compound **1**; **(C)**: compound **2**; (D) compound **3**; **(E)**: extract; **(F)**: gentamicin.

In this investigation, the crude extract and compounds **1**–**8,** isolated from the methanol extract of the aerial parts of S. *muricatum* were used to determine % of biofilm reduction at ¼ and ½ MIC (µM) values against the tested methicillin-resistant *Staphylococcus aureus* (ATCC 43300), using the broth dilution method (Fig 3). The results showed that compounds **1**, **2**, and **3** can reduce biofilm formation in a percentage range from 62.99 to 66.90 at ¼ MIC, and in a range of 70.11 to 75.09% at **½** MIC (Table 3). Gentamicin was used as a positive control (% of biofilm reduction at ¼ and ½ MIC = 80.00 and 91.33%, respectively) (Table 3).

**Table 3:** Effect of the crude extract, and tested compounds on biofilm formation by MRSA.

Tested compound	% of biofilm reduction at ¼ MIC	% of biofilm reduction at ½ MIC
1	65.48	70.11
2	62.99	75.09
3	66.90	70.11
4	7.00	17.44
5	9.00	15.30
6	15.30	65.84
7	5.60	23.84
8	12.10	64.77
Extract	8.90	41.64
Gentamicin	80.00	91.33

The GraphPad Prism 9 software was used to calculate % of biofilm reduction at ¼ and ½ the minimum inhibitory concentration MIC (µM) values. The concentration of each sample was transformed to log10. All experiments were performed at least three times. *p < 0.05, Student’s *t*-test.

### 3.4. Protein-protein interaction network

#### 3.4.1. *Therapeutic targets for MRSA infections:*

Based on our experimental findings, we compiled a dataset of 51 target proteins associated with methicillin-resistant *Staphylococcus aureus* (MRSA) infections. These targets were meticulously curated from publicly available databases, including NCBI-GEO and PharmGKB, and further validated through our experimental investigations. As depicted in S5 Table in S1 File, these proteins play pivotal roles in MRSA pathogenesis and antibacterial resistance mechanisms, making them potential therapeutic targets for drug development. This analysis strengthens our understanding of MRSA-associated pathways and highlights key molecular players that could be modulated for improved therapeutic strategies.

#### 3.4.2. STITCH database correlation of MRSA targets and Solanum muricatum compounds.

**Fig 4:**
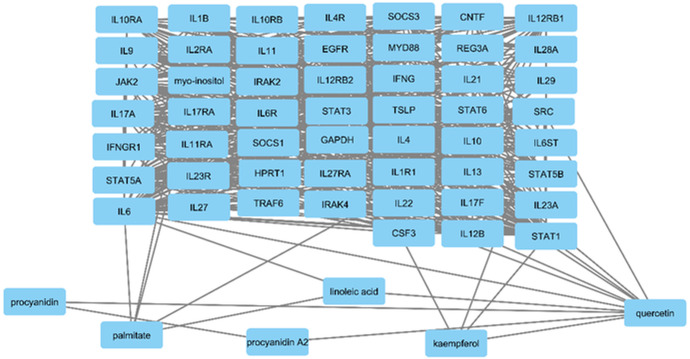
STITCH analysis of interactions between *S. muricatum* phytochemicals (kaempferol, quercetin, procyanidin A2, palmitic acid, linoleic acid) and methicillin-resistant *Staphylococcus aureus* (MRSA) targets. STITCH = Search tool for interacting chemicals.

To investigate the molecular interactions between bioactive compounds from *S. muricatum* and MRSA-related target proteins, we employed the STITCH database for network analysis. As shown in Fig 4, this analysis examined interactions between the key phytochemicals, including quercetin, kaempferol, procyanidin A2, linoleic acid, and palmitic acid, with the curated MRSA protein targets. The analysis revealed a high degree of connectivity, indicating that these phytochemicals engage with manifold immune-regulatory and antibacterial resistance-related proteins. Notably, quercetin and kaempferol exhibited strong interactions with key signaling molecules such as STAT3, IL6, and IL17, which are implicated in MRSA immune evasion and inflammatory responses. Similarly, procyanidins and linoleic acid demonstrated significant interactions with cytokine receptors (IL10RA, IL4R, IL2RA) and transcription factors that modulate immune responses. This analysis underscores the potential synergistic activity of *S. muricatum* compounds in modulating MRSA-associated molecular pathways. The results highlight their ability to target multiple cellular mechanisms simultaneously, thereby enhancing the anti-*Staphylococcus* efficacy and reducing the likelihood of resistance development.

#### 3.4.3. *Protein network construction for MRSA interaction with Solanum muricatum.*

**Fig 5:**
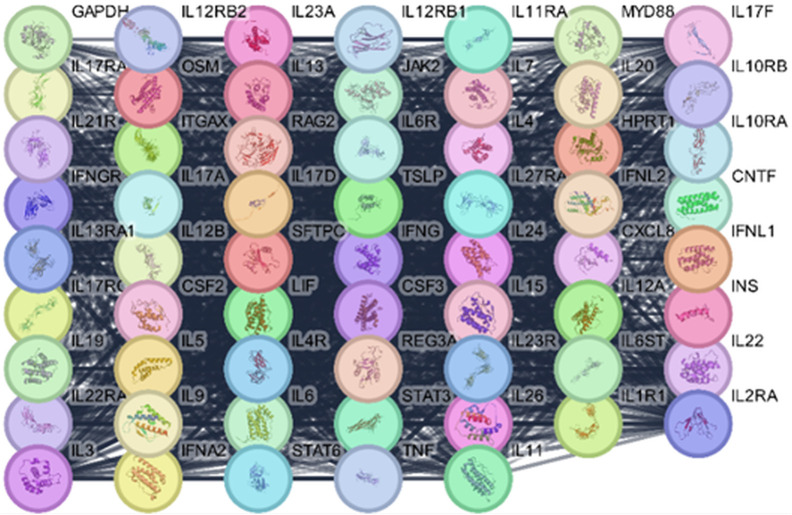
STRING network of protein–protein interactions (PPIs) among MRSA-related targets and *S. muricatum* bioactive compounds generated using Cytoscape v3.10.3. STRING = Search tool for the retrieval of interacting genes/proteins; PPI = protein–protein interaction.

To construct a comprehensive protein-protein interaction (PPI) network that elucidates the interactions between MRSA-related proteins and *S. muricatum* bioactive compounds, we utilized the STRING database version 12.0. This analysis aimed to uncover key molecular targets influenced by phytochemicals and their potential role in modulating MRSA pathogenesis. The PPI network visualization, as depicted in Fig 5, was developed using Cytoscape version 3.10.3, where protein-protein interactions (PPIs) and compound-protein interactions were integrated. The network consists of 61 nodes and 1,250 interaction linkages, demonstrating an average node connectivity of 41.67. These interactions indicate strong functional correlations between MRSA-related targets and the phytochemicals identified from *S. muricatum*. From the analysis, the phytochemical compounds emerged as central bioactive compounds with extensive interactions across immune-regulatory proteins and MRSA-associated inflammatory mediators, including IL17RA, STAT3, IL6, IL12RB1, and TNF. The network highlights how these compounds might influence cytokine signaling, immune modulation, and bacterial persistence mechanisms. Furthermore, they also demonstrated interactions with proteins related to metabolic and immune response pathways, reinforcing their potential anti-*Staphylococcus* effects. The intricate connectivity and interactions further validate the multi-target potential of *S. muricatum* compounds, offering a promising avenue for novel antibacterial drug development.

#### 3.4.4. Identification of hub genes in the MRSA-associated PPI network.

**Fig 6:**
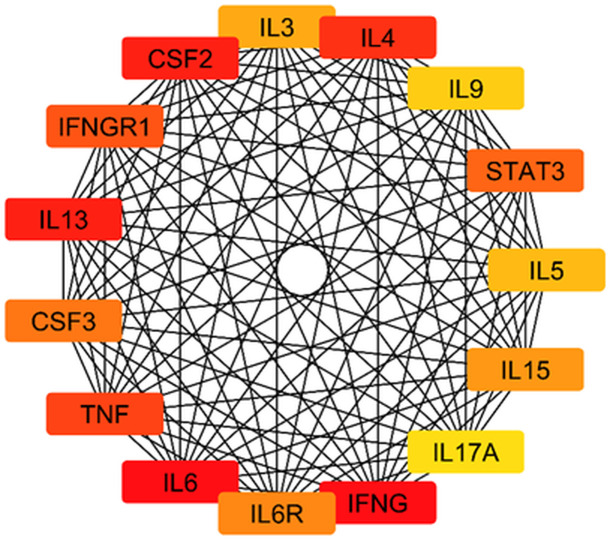
Identification of hub genes in the MRSA-associated protein-protein interaction (PPI) network. The CytoHubba analysis identified key hub genes within the MRSA-related protein-protein interaction (PPI) network, highlighting their high connectivity and functional significance.

The CytoHubba plugin was used to identify key hub genes within the protein-protein interaction (PPI) network related to the potential antibacterial effects of *Solanum muricatum* bioactive compounds against MRSA. As illustrated in Fig 6, these hub genes exhibit high connectivity, making them critical to the network and valuable targets for further exploration. The analysis highlighted several immune and inflammation-related genes, including IL6, IL4, IL9, STAT3, IL17A, TNF, and IFNG, all of which play significant roles in host-defense mechanisms. IL6 and TNF are central mediators of inflammation and have been linked to the host immune response against bacterial infections, including MRSA. STAT3 and IFNG are key regulators of immune signaling, particularly in modulating inflammatory pathways and antibacterial immunity. IL4, IL9, and IL17A contribute to cytokine-mediated immune responses, influencing both innate and adaptive immunity during MRSA infections. The strong connectivity among these hub genes suggests a dynamic immune response to MRSA infection, where *S. muricatum* compounds may modulate inflammatory pathways, cytokine signaling, and immune cell activation. This potential mechanism could enhance the innate immune response of the host, while interfering with immune evasion strategies. These findings provide a deeper understanding of protein interactions relevant to MRSA pathogenesis and suggest that targeting these highly connected genes could serve as an effective therapeutic strategy. The ability of *S. muricatum* compounds to influence these pathways reinforces their potential as novel anti-*Staphylococcus* agents for combating MRSA infections.

### 3.5. Analysis of enriched gene ontology (GO) terms

**Fig 7:**
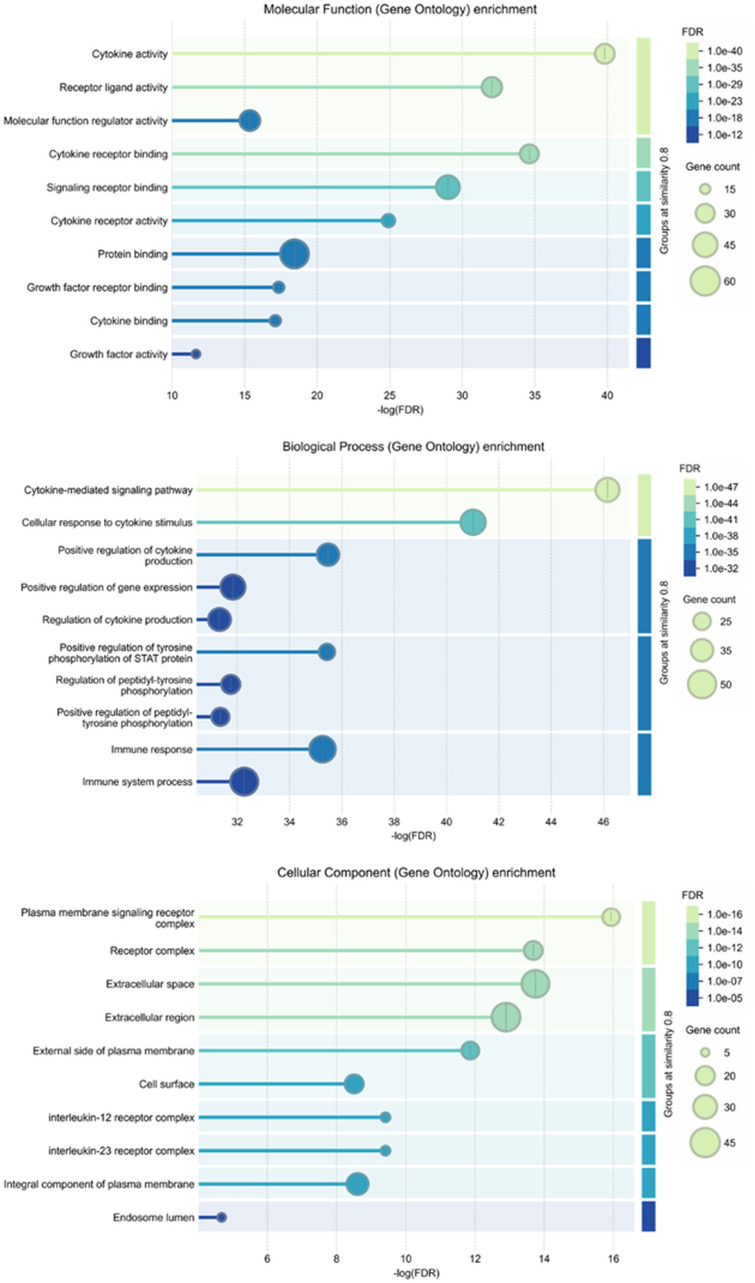
Gene ontology (GO) enrichment analysis of MRSA-associated targets and *Solanum muricatum* bioactive compounds.

To explore the molecular mechanisms underlying the anti-*Staphylococcus* potential of *S. muricatum* bioactive compounds against MRSA, a gene ontology (GO) enrichment analysis was performed using ShinyGO v0.80. The results identified key biological processes, cellular components, and molecular functions associated with MRSA pathogenesis and immune response modulation. In the biological process (BP) category, pathways such as “*Cytokine-mediated signaling*”, “*Regulation of cytokine production*”, and “*Immune system process*” were significantly enriched. These processes play essential roles in coordinating immune responses, suggesting that the bioactive compounds may contribute to immune modulation and inflammation control in the presence of MRSA infections. The cellular component (CC) analysis identified significant terms, including “*Plasma membrane signaling receptor complex*”, “*Extracellular space*”, and “*Endosome lumen*”, indicating that the targeted proteins are predominantly involved in membrane-associated and extracellular immune functions. These components are crucial for pathogen recognition, immune signaling, and cellular communication, pointing to potential mechanisms of anti-*Staphylococcus* activity. For molecular function (MF) enrichment, notable terms such as “*Cytokine activity*”, “*Receptor ligand activity*”, and “*Cytokine receptor binding*” were highlighted. These findings suggest that the studied compounds may play a role in modulating cytokine signaling and receptor interactions, potentially influencing the immune response of the host against MRSA. Overall, these insights provide a deeper understanding of how *S. muricatum* compounds might interact with immune pathways and antibacterial defense mechanisms. The visual representations in Fig 7 illustrate the significantly enriched GO terms across BP, CC, and MF categories. Further details, including statistical significance and specific GO terms, are documented in the Supplementary S6 Table in S1 File, supporting the potential therapeutic applications of these bioactive compounds against MRSA infections.

### 3.6. Analysis of key KEGG pathways

**Fig 8:**
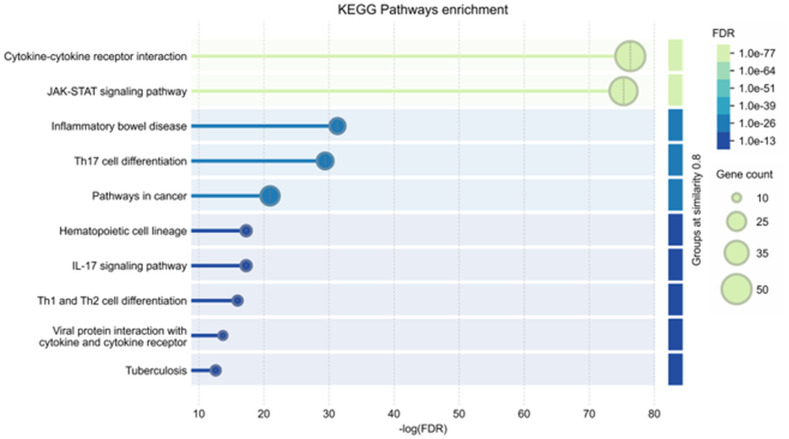
Kyoto Encyclopedia of Genes and Genomes (KEGG) pathway enrichment analysis of targets modulated by *S. muricatum* compounds against MRSA.

The KEGG pathway enrichment analysis was conducted to explore the biological pathways influenced by bioactive compounds from *S. muricatum* in relation to MRSA infections. This analysis provides a deeper understanding of the molecular mechanisms through which these compounds may exert anti-*Staphylococcus* activity. As shown in Fig 8, several immune-related and signaling pathways were significantly enriched, including cytokine-cytokine receptor interactions, JAK-STAT signaling, IL-17 signaling, and Th17 cell differentiation. These pathways are essential for immune modulation and inflammatory responses, suggesting that the identified compounds may contribute to enhancing host-defense mechanisms against MRSA by regulating immune signaling. Additionally, the involvement of pathways such as hematopoietic cell lineage and Th1/Th2 cell differentiation indicates a possible role in adaptive immune system regulation, which could improve the ability to respond to MRSA infections. Other enriched pathways, such as tuberculosis and viral protein interactions with cytokine receptors, suggest broader implications for pathogen defense and immune activation. These findings, further detailed in the Supplementary S7 Table in S1 File, highlight the potential of *S. muricatum* compounds to interact with host immune pathways to counteract MRSA infections.

**Fig 9:**
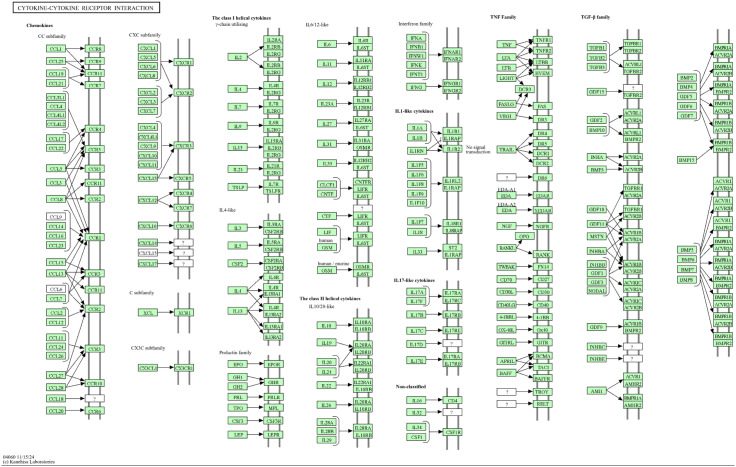
KEGG pathway of cytokine-cytokine receptor interaction in MRSA infection. The enrichment of these pathways indicates that bioactive compounds from *Solanum muricatum* may modulate immune responses by restoring cytokine balance and enhancing host-defense mechanisms.

The KEGG pathway analysis identified the cytokine-cytokine receptor interaction as a key molecular network influenced by *S. muricatum* bioactive compounds in their potential role against MRSA infections. This pathway comprises pro-inflammatory cytokines, immune signaling molecules, and receptor-mediated interactions, all of which are essential in regulating host-immune responses to bacterial infections. As illustrated in Fig 9, several immune-related proteins and signaling components were found to be significantly enriched. The analysis suggests that MRSA may disrupt immune defense mechanisms by modulating cytokine interactions, altering inflammatory pathways, and inhibiting opsonization processes. The presence of *S. muricatum* bioactives could potentially counteract these effects by rebalancing immune signaling, promoting inflammation where necessary, and enhancing the ability to eliminate MRSA. These findings offer valuable insights into the molecular mechanisms underlying the anti-*Staphylococcus* potential of *S. muricatum*. The ability of its bioactive compounds to modulate immune signaling and prevent immune evasion tactics underscores its potential as a natural source for new therapeutic interventions. By targeting host-pathogen interactions at the molecular level, these compounds may contribute to the development of effective strategies for combating MRSA infections.

### 3.7. Molecular docking analysis of Solanum muricatum compounds against 3’,5“-aminoglycoside phosphotransferase type IIIa (APH(3’)-IIIa)

**Fig 10:**
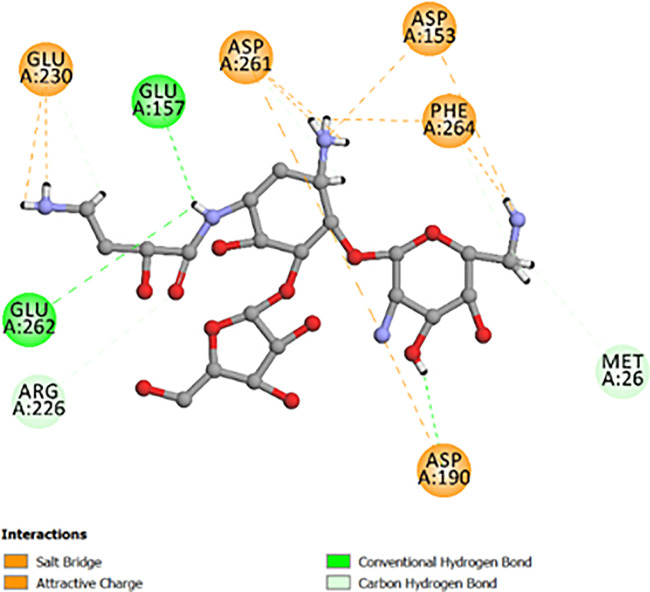
Molecular interaction analysis of butirosin A (co-ligand) with APH(3’)-IIIa.

Aminoglycoside resistance in MRSA is largely mediated by 3’,5“-aminoglycoside phosphotransferase type IIIa (APH(3’)-IIIa), an enzyme responsible for phosphorylating aminoglycoside antibiotics, leading to their inactivation. This modification prevents aminoglycosides such as kanamycin, neomycin, and tobramycin from effectively binding to bacterial ribosomes, thereby reducing their bactericidal activity. The presence of APH(3’)-IIIa in MRSA significantly complicates treatment, as it allows the bacteria to evade manifold classes of antibiotics. Our network analysis integrates APH(3’)-IIIa within the broader MRSA resistance landscape, highlighting its interactions with immune-related pathways, cytokine signaling networks, and antibacterial resistance mechanisms. By mapping these interactions, we can better understand how MRSA modulates both bacterial defense and host immune evasion strategies to maintain its survival. Additionally, C3, a key component of the complement system, plays a crucial role in the host defense against MRSA infections. Complement activation, particularly through membrane attack complexes (MACs) and opsonization, can enhance bacterial clearance independently of antibiotic efficacy. This suggests that while APH(3’)-IIIa contributes to antibiotic resistance, activation of complement pathways may still provide an effective immune defense mechanism against MRSA infections. Our study indicates that bioactive compounds from *S. muricatum* could potentially modulate these resistance mechanisms, either by inhibiting APH(3’)-IIIa directly or by enhancing immune responses through complement activation and cytokine signaling. Understanding these interactions provides a foundation for developing novel therapeutic strategies that can circumvent antibiotic resistance and improve MRSA clearance. Molecular docking was conducted to evaluate the binding interactions of bioactive compounds from *S. muricatum* with 3’,5”-aminoglycoside phosphotransferase type IIIa (APH(3’)-IIIa). The aminoglycoside phosphotransferase APH(3’)-IIIa (PDB ID: 3TM0) was selected due to its central role in mediating aminoglycoside resistance in MRSA by phosphorylating antibiotics like kanamycin and gentamicin. This target is well validated in antibacterial resistance studies. The docking studies were performed using OPLS-AA (optimized potentials for liquid simulations – all atom) force field, ensuring accurate energy minimization and structural refinement of receptor-ligand complexes. The binding energies (kcal/mol) and RMSD (Root-mean-square deviation) refinement values for all tested ligands are summarized in S8 Table in S1 File, which provides an overview of ligand stability and interaction strength. Among the tested compounds, kaempferol 3-*O*-gentiobioside (**1**) exhibited the highest binding affinity (−8.102 kcal/mol, RMSD 1.254), suggesting a strong interaction within the APH(3’)-IIIa active site. The second and third most potent ligands, kaempferol 3-sambubioside (**2**) (−7.984 kcal/mol, RMSD 1.369) and quercetin 3-rhamnoside (**3**) (−7.872 kcal/mol, RMSD 1.458), also demonstrated notable binding interactions, reinforcing their potential as APH(3’)-IIIa inhibitors. The docking poses of butirosin A (co-ligand) and gentamicin (reference ligand) within the APH(3’)-IIIa active site serve as controls for assessing ligand interactions. Butirosin A displayed a variety of crucial interactions with Asp153, Asp190, Asp261, Glu157, Phe264, Met26, and Glu230, stabilizing its positioning within the catalytic pocket (Fig 10).

**Fig 11:**
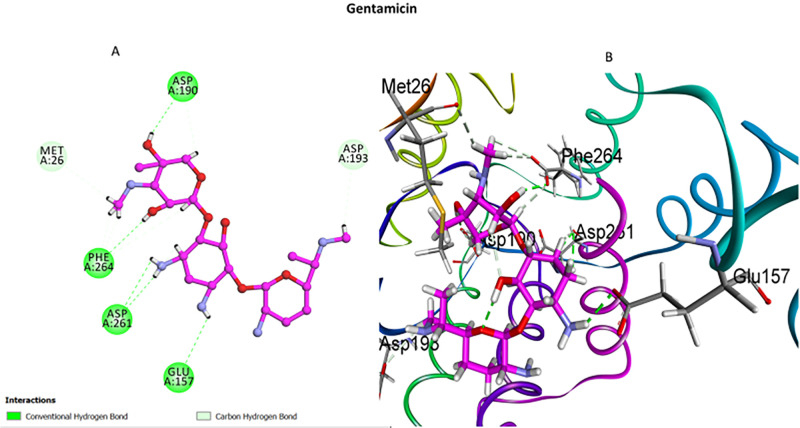
Molecular interaction analysis of gentamicin (reference ligand) with APH(3’)-IIIa. **(A)**: 2D docking map within APH(3’)-IIIa demonstrates strong hydrogen bonding interactions with Asp190, Asp261, Phe264, and Met26, ensuring its stable binding to the enzyme; **(B)**: 3D visualization illustrates the orientation within the active site pocket, showing alignment with crucial catalytic residues involved in aminoglycoside phosphorylation and resistance mechanisms.

Similarly, gentamicin formed hydrogen bonds with Asp190, Asp261, Phe264, and Met26, mimicking the interaction profile of butirosin A (Fig 11). The overlap between these binding patterns validates the docking methodology and confirms their role as effective reference ligands for assessing novel inhibitors.

Although compound **1** (kaempferol 3-*O*-gentiobioside) demonstrated the most potent *in vitro* anti-MRSA activity (MIC = 8.3 µM), compound **2** (kaempferol 3-*O*-sambubioside) also exhibited comparable efficacy (MIC = 10.2 µM), with only a modest difference in activity. Given this minimal variation in MIC values, both compounds were considered promising candidates. However, compound **1** was prioritized for further docking analysis due to its superior binding affinity. These results indicated a more favorable and stable interaction profile than compound **2**.

**Fig 12:**
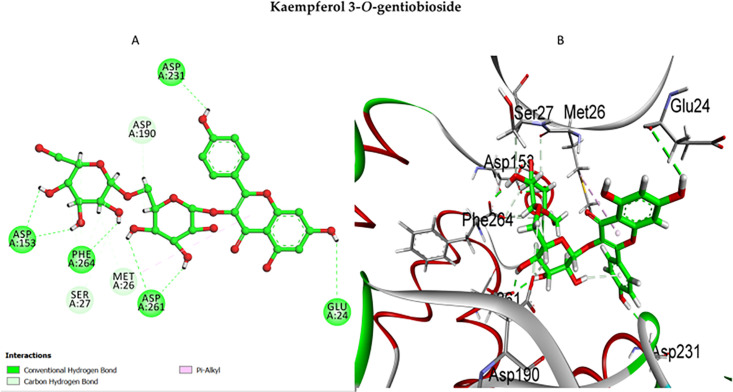
Molecular docking analysis of kaempferol 3-*O*-gentiobioside (1) with APH(3’)-IIIa. **(A)**: 2D docking map of kaempferol 3-*O*-gentiobioside **(1)**, revealing strong hydrogen bonding interactions with Asp153, Asp190, Asp231, Glu24, and Met26, ensuring its tight binding within the APH(3’)-IIIa active site; **(B)**: 3D visualization confirms that compound 1 aligns well with the catalytic site, exhibiting a binding pose comparable to that of the co-ligand (butirosin **A**).

The docking pose of kaempferol 3-*O*-gentiobioside (**1**) within the APH(3’)-IIIa binding pocket revealed a high-affinity interaction profile, positioning it among the most promising inhibitors. As shown in Fig 12, the ligand formed various hydrogen bonds with Asp153, Asp190, Asp231, Glu24, Phe264 and Met26, ensuring strong interaction with the catalytic domain. Additionally, the π-alkyl interaction with Met26 facilitated aromatic stacking, contributing to hydrophobic stabilization within the binding site. The 3D visualization confirms that compound **1** effectively aligns within the APH(3’)-IIIa active site, maintaining a binding pose comparable to that of butirosin A.

Docking results of the isolated compounds, reference antibiotic (kanamycin A), and co-crystallized ligand (butirosin A) are summarized in Table 4.

**Table 4:** Summary of molecular docking results for isolated compounds and control ligands against APH (3′)-IIIa (PDB 3TM0).

Compound	Docking Score (kcal/mol)	RMSD (Å)	No. of H-bonds	Key Interacting Residues	Interaction Type	Remarks
**1** (Kaempferol 3-*O*-gentiobioside)	−10.3	1.28	6	Lys33, Asp190, Asn195, Glu208	H-bond + π-stacking	Highest affinity; stable complex in MD
**2** (Kaempferol 3-*O*-sambubioside)	−9.7	1.35	5	Glu208, Lys44, Ser185	H-bond + electrostatic	Strong binding energy comparable to **1**
**3 (**Quercetin 3-*O*-rhamnoside)	−9.1	1.42	4	Asp190, Lys33, Tyr42	H-bond + hydrophobic	Moderate stability
**4** (Procyanidin A2)	−8.6	1.56	5	Asn195, Lys44, Glu208	H-bond + π-stacking	Good binding energy
**5** (Procyanidin A2 3-*O*-glucoside)	−8.2	1.6	4	Asp190, Ser185	H-bond	Moderate binding
**6** (Galactopyranoside derivative)	−7.9	1.78	3	Lys44, Asn195	H-bond	Weak interaction
**7** (Palmitic acid)	−6.5	1.94	2	Glu208, Leu52	Hydrophobic	Low affinity
**8** (Linoleic acid)	−6.2	1.99	2	Ala51, Lys44	Hydrophobic	Low affinity
Control (Kanamycin A)	−8.8	1.31	4	Lys33, Asp190, Asn195	H-bond	Reference ligand
Co-crystallized ligand (Butirosin A)	−9.0	1.25	4	Lys33, Glu208	H-bond	Used for active site validation

The integrative docking analysis suggests that bioactive compounds from *S. muricatum* could serve as potential inhibitors of APH(3’)-IIIa, thereby restoring aminoglycoside efficacy against MRSA infections. The structural interactions observed in kaempferol 3-gentiobioside (**1**), kaempferol 3-sambubioside (**2**), and quercetin 3-rhamnoside (**3**) indicate their ability to disrupt the enzymatic phosphorylation of aminoglycosides, a crucial step in MRSA resistance. The combination of hydrogen bonding, salt bridge formation, and hydrophobic interactions ensures that these compounds remain tightly bound within the APH (S’)-IIIa active site, reinforcing their potential as antibiotic adjuvants. These findings align with our experimental validation, offering a promising avenue for the development of plant-based inhibitors to counteract MRSA aminoglycoside resistance. Based on this favorable interaction profile, kaempferol 3-gentiobioside (1) was advanced to molecular dynamics simulation, which further confirmed the stability of its binding.

### 3.8. Molecular dynamics (MD) simulation analysis of kaempferol 3-O-gentiobioside (1) against 3’,5“-aminoglycoside phosphotransferase type IIIa

**Fig 13:**
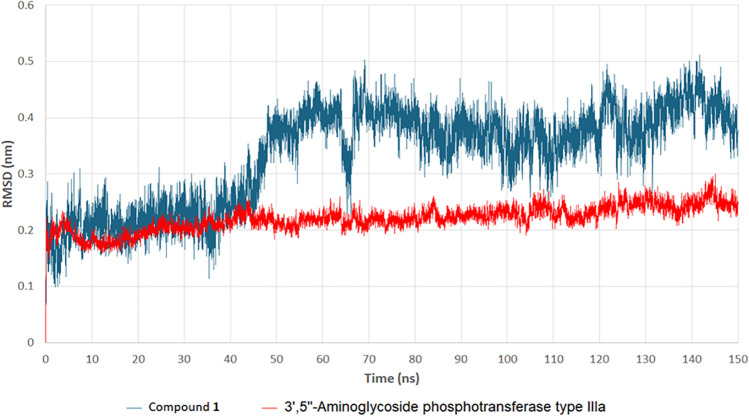
RMSD (Root-mean-square deviation) analysis of compound 1 and 3’,5“-aminoglycoside phosphotransferase type IIIa (APH(3’)-IIIa) over 150 ns. The structural stability of APH(3’)-IIIa (red line) and compound **1** (blue line) over the simulation time.

To evaluate the stability, binding affinity, and dynamic behavior of kaempferol 3-*O*-gentiobioside (**1**) within the active site of 3’,5“-aminoglycoside phosphotransferase type IIIa (APH(3’)-IIIa), a 150 ns molecular dynamics (MD) simulation was conducted. Several critical parameters, including root mean square deviation (RMSD), hydrogen bonding analysis, radius of gyration (Rg), and potential energy, were assessed to determine the structural integrity and stability of the protein-ligand complex. The RMSD plot (Fig 13) illustrates the structural deviations of both compound **1** and APH(3’)-IIIa over time. The protein RMSD (red line) remained stable throughout the simulation, fluctuating between 0.15 and 0.25 nm, suggesting that the enzyme maintained its structural integrity during the simulation. Conversely, compound **1** (blue line) exhibited higher fluctuations, particularly after 50 ns, with RMSD values ranging from 0.2 to 0.5 nm. These variations indicate a degree of conformational flexibility within the binding pocket, which may influence the binding efficiency. Despite these fluctuations, compound **1** remained stably bound to the active site of APH(3’)-IIIa, suggesting strong ligand-enzyme interactions.

**Fig 14:**
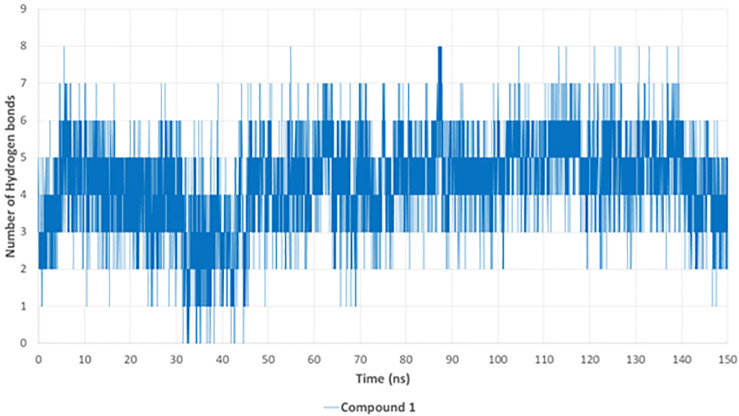
Hydrogen bond analysis of compound 1 with APH(3’)-IIIa over 150 ns.

The hydrogen bond analysis (Fig 14) provides insight into the binding stability of compound **1** throughout the MD simulation. On average, three to six hydrogen bonds were maintained consistently over the simulation time, with intermittent peaks reaching eight hydrogen bonds. These persistent interactions reinforce the strong binding affinity of compound **1** within the APH(3’)-IIIa active site. The fluctuations in hydrogen bonding suggest minor conformational adjustments, but the consistent presence of various hydrogen bonds indicates a stable interaction network between compound **1** and key catalytic residues.

**Fig 15:**
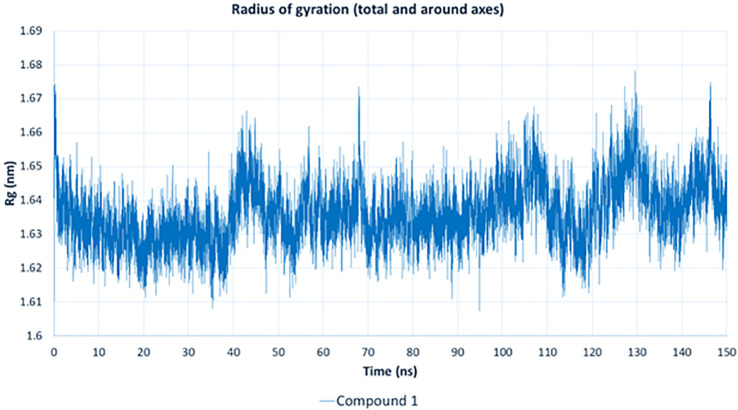
Radius of gyration (Rg) analysis of the APH(3’)-IIIa-compound 1 complex over 150 ns.

The radius of gyration (Rg) analysis (Fig 15) assesses the structural compactness of the complex of compound **1** with APH(3’)-IIIa. Throughout the 150 ns simulation, the Rg values remained stable, fluctuating between 1.61 and 1.67 nm, indicating that the protein did not undergo significant structural expansion or collapse. This stability suggests that the binding of compound **1** did not disrupt the overall protein structure, allowing the enzyme to retain its functional conformation while maintaining ligand interactions.

**Fig 16:**
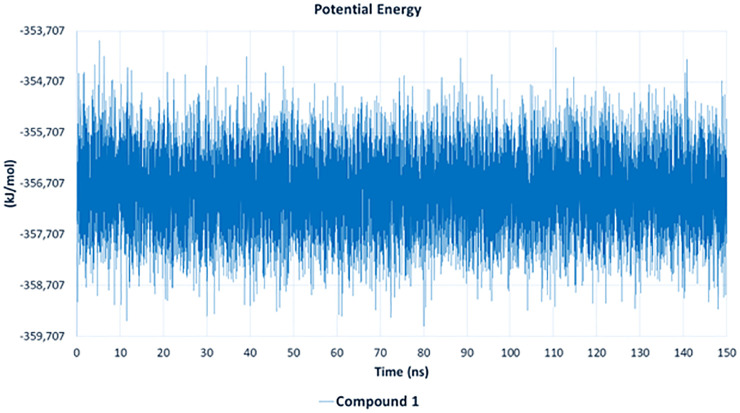
Potential-energy analysis of the complex of APH(3’) IIIa with compound 1, over 150 ns.

The potential energy plot (Fig 16) highlights the thermodynamic stability of the complex of compound **1** with APH(3’)-IIIa. The potential energy values fluctuated minimally, maintaining a stable range of approximately −356,000 to −358,500 kJ/mol throughout the simulation. This consistent energy profile further validates the stability of the protein-ligand system, confirming that compound **1** remains stably bound without causing significant energetic perturbations.

The MD simulation results confirm that compound **1** exhibits strong and stable binding with APH(3’)-IIIa, demonstrating minimal RMSD fluctuations, consistent hydrogen bonding, stable protein compactness (Rg), and low potential energy, indicating a thermodynamically stable interaction. These findings suggest that compound **1** effectively disrupts aminoglycoside phosphorylation, potentially restoring antibiotic efficacy against MRSA.

## 4. Conclusion

The phytochemical investigation of *S. muricatum* aerial parts led to the isolation of eight known compounds, predominantly flavonoid derivatives. Among these, compounds **1**–**3** demonstrated notable antibacterial activity against MRSA, with compound **1** (kaempferol 3-gentiobioside) showing the highest potency (MIC = 8.3 µM) and significant biofilm inhibition. Synergistic effects were observed both among the active compounds and in combination with gentamicin, enhancing their anti-*Staphylococcus* potential. Complementary computational analyses, including network pharmacology, molecular docking, and molecular dynamics simulations, confirmed the strong and stable interaction of compound **1** with the resistance enzyme APH(3’)-IIIa, suggesting a mechanism by which it may overcome aminoglycoside resistance in MRSA. These findings collectively highlight *S. muricatum* as a promising source of bioactive compounds with potential for the development of new plant-derived therapeutics targeting antibiotic-resistant pathogens.

## Supporting information

S1 File NMR spectra (¹H and DEPT-Q) and NMR data tables for compounds 1–8, together with the MRSA-related protein list, GO and KEGG enrichment entries, and docking binding-energy data. (DOCX)

## Abbreviations

MIC: Minimum inhibitory concentration
MBC: Minimum bactericidal concentration
FICI: Fractional inhibitory concentration index
MD: Molecular dynamics
RMSD: Root-mean-square deviation
RMSF: Root-mean-square fluctuation
Rg: Radius of gyration
H-bond: Hydrogen bond
GO: Gene ontology
KEGG: Kyoto Encyclopedia of Genes and Genomes
PPI: Protein–protein interaction
STITCH: Search tool for interacting chemicals
STRING: Search tool for the retrieval of interacting genes/proteins
