# Supplementary material for: Antibacterial efficacy of Solanum muricatum aiton metabolites against methicillin-resistant staphylococcus aureus: Insights into bioactive compounds and molecular mechanisms
Source: PLoS One. 2025 Dec 26;20(12):e0338733. doi: 10.1371/journal.pone.0338733 (PMC12742761; doi:10.1371/journal.pone.0338733)
Supplement: S1 File — (DOCX) [file pone.0338733.s001.docx]

***Antibacterial Efficacy of Solanum muricatum Aiton Metabolites Against Methicillin-Resistant Staphylococcus aureus: Insights into Bioactive Compounds and Molecular Mechanisms***

**Sarah Samir** **^‡1^, [Fathy A. Behery](https://pubmed.ncbi.nlm.nih.gov/?sort=date&term=Behery+FA&cauthor_id=33673168)^‡2,3^,Mohamed A. Zarka^4^, Ruqaiah I. Bedaiwi^5^, Hesham A. Abou-Zied^6^, Usama Ramadan Abdelmohsen^7,8*^, Rehab Mahmoud Abd El-Baky^9,10^, Mohamed A. Mawhoup^10^, Mai Mahrous^10^, Reem E. S. Abdelnaem^7^, Gerhard Bringmann^11^, Abeer H. Elmaidomy^12,*^**

^1^Departement of Pharmacognosy, Faculty of Pharmacy, Misr University for Science and Technology (MUST), ^6^th October city, , Giza, 12566, Egypt. [Sarah.samir@must.edu.eg](mailto:Sarah.samir@must.edu.eg)

^2^Department of Pharmacognosy, Faculty of Pharmacy, Mansoura University, Mansoura 35516, Egypt. fathybehery@mans.edu.eg

^3^Department of Pharmacy, College of Pharmacy, Nursing and Medical Sciences, Riyadh Elm University, Riyadh 11681, Saudi Arabia^4^Department of Pharmacognosy, College of Pharmacy, The Islamic University, Najaf, Iraq. dr.m.abdulaal84@iunajaf.edu.iq

^5^Department of Medical Laboratory Technology, Faculty of Applied Medical Sciences, 71491 University of Tabuk, Tabuk, Saudi Arabia. rbedaiwi@ut.edu.sa

^6^Department of Medicinal Chemistry, Faculty of Pharmacy, Deraya University, Minia 61111, Egypt. hisham.alaa@deraya.edu.eg

^7^Deraya Center for Scientific Research, Deraya University, Minia 61111, Egypt. usama.ramadan@mu.edu.eg, [reem.esamelden_1220445@student.deraya.edu.eg](mailto:reem.esamelden_1220445@student.deraya.edu.eg)

^8^Department of Pharmacognosy, Faculty of Pharmacy, Minia University, 61519 Minia

^9^Department of Microbiology and Immunology, Faculty of Pharmacy, Minia University, Minia 61519, Egypt. rehab.mahmoud@mu.edu.eg

^10^Department of Microbiology and Immunology, Faculty of Pharmacy, Deraya University, Minia 11566, Egypt. [rehab.mahmoud@mu.edu.eg](mailto:rehab.mahmoud@mu.edu.eg), [mohamed.mawhoup@deraya.edu.eg](mailto:mohamed.mawhoup@deraya.edu.eg), [mai.mahrous@deraya.edu.eg](mailto:mai.mahrous@deraya.edu.eg),

^11^Institute of Organic Chemistry, University of Würzburg, Am Hubland, 97074 Würzburg, Germany. [gerhard.bringmann@uni-wuerzburg.de](mailto:gerhard.bringmann@uni-wuerzburg.de)

^12^Department of Pharmacognosy, Faculty of Pharmacy, Beni-Suef University, Beni-Suef 62514, Egypt. [Abeer011150@pharm.bsu.edu.eg](mailto:Abeer011150@pharm.bsu.edu.eg)

*****Correspondence: authors: usama.ramadan@mu.edu.eg (U.R.A.), [Abeer011150@pharm.bsu.edu.eg](mailto:Abeer011150@pharm.bsu.edu.eg) (A.H.E.).

^‡^ These authors contributed equally to this work.

**Figure S1.** ^1^H NMR spectrum of compound **1 (**kaempferol 3-*O*-gentiobioside**)** measured in CD_3_OD at 400 MHz.


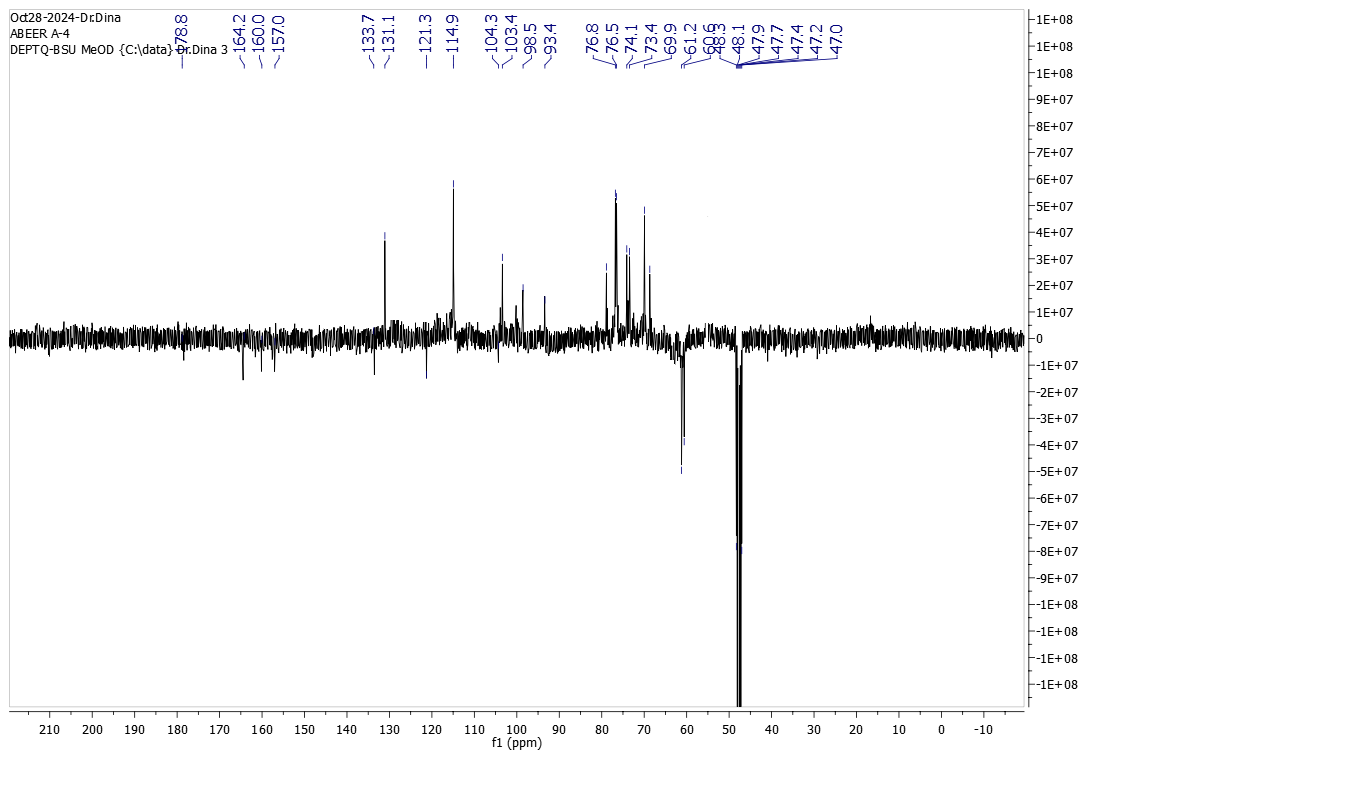


**Figure S2.** DEPT-Q NMR spectrum of compound **1 (**kaempferol 3-*O*-gentiobioside**)** measured in CD_3_OD at 100 MHz.

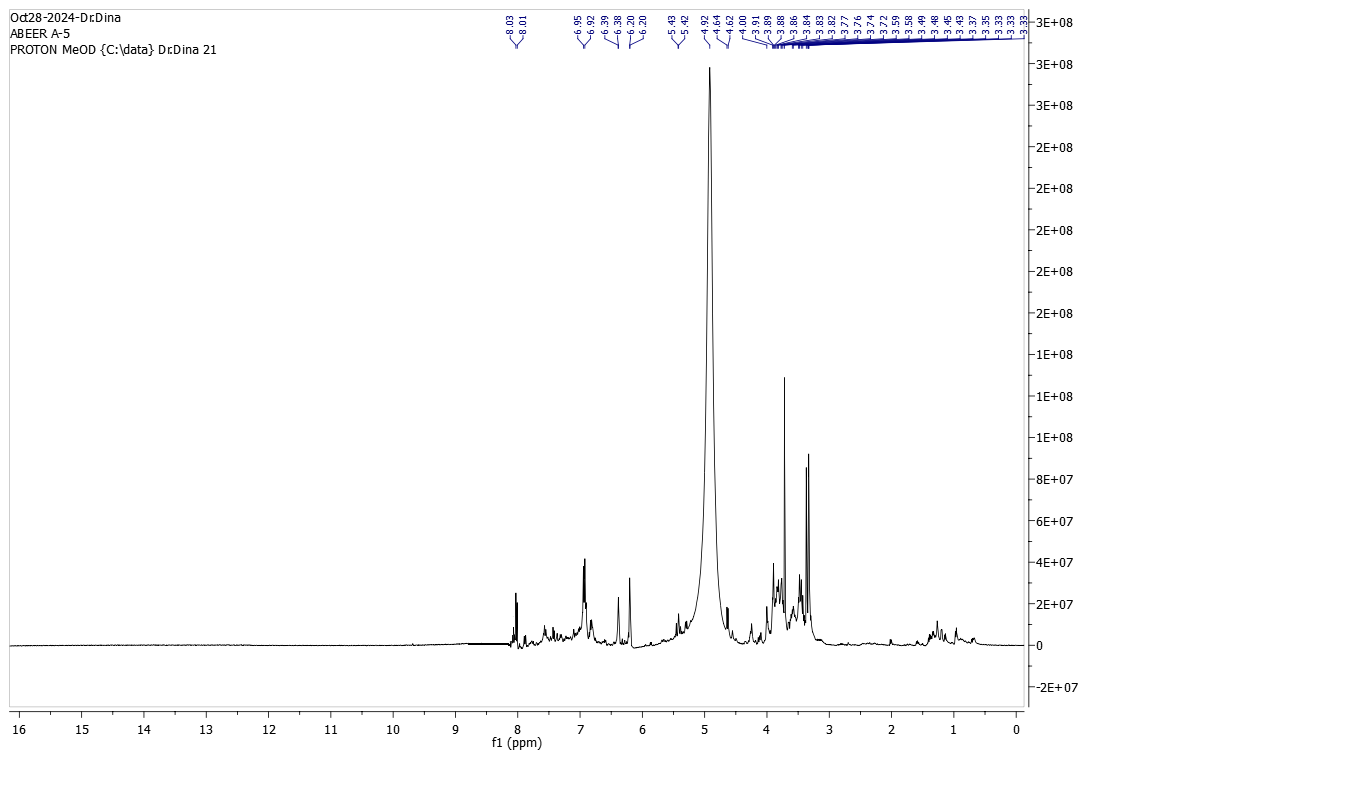


**Figure S3.** ^1^H NMR spectrum of compound **2** (kaempferol 3-*O*-sambubioside) measured in CD_3_OD at 400 MHz.


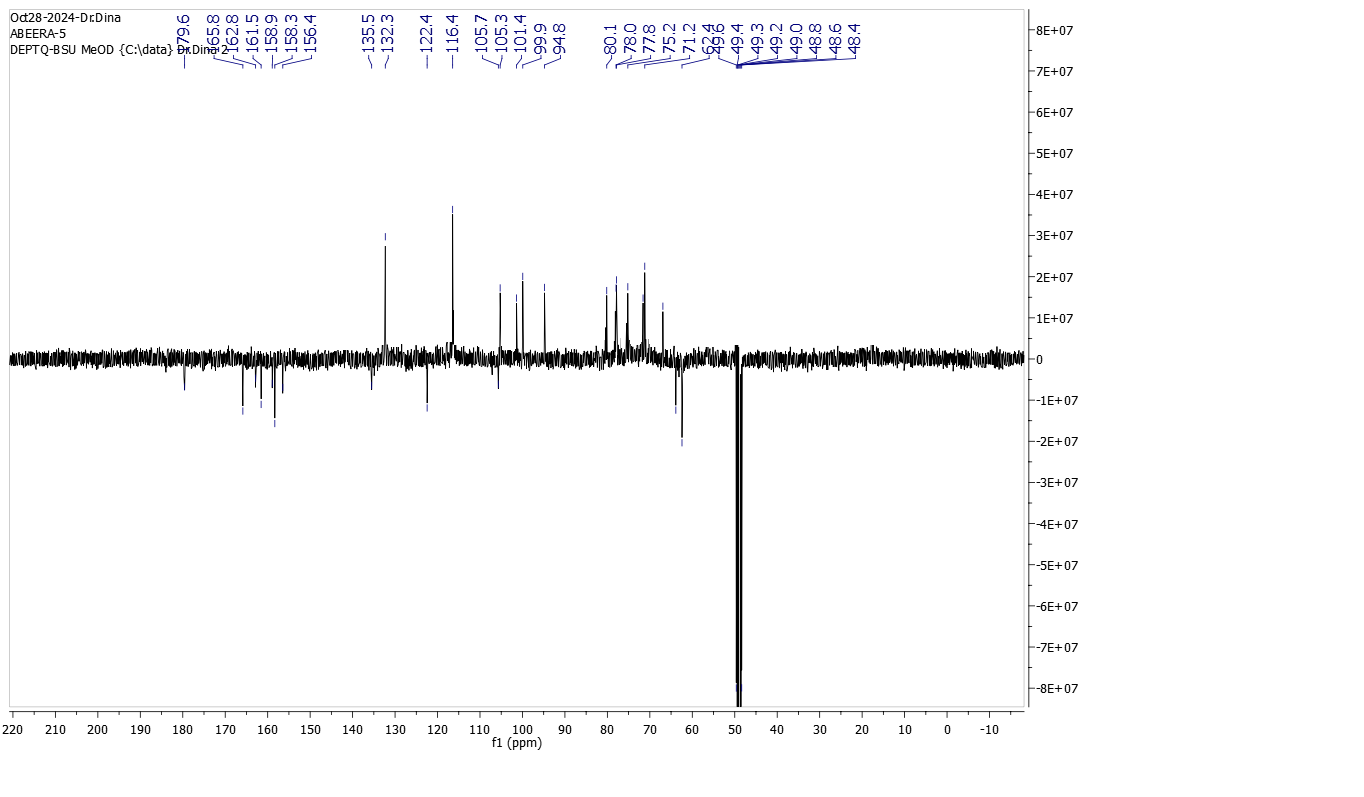


**Figure S4.** DEPT-Q NMR spectrum of compound **2** (kaempferol 3-*O*-sambubioside) measured in CD_3_OD at 100 MHz.

**Table S1.** DEPT-Q (400 MHz) and ^1^H (100 MHz) NMR data for compounds **1** and **2** in CD_3_OD; carbon multiplicities were determined by DEPT-Q experiments.

|  | Position | 1 | | 2 | |
| --- | --- | --- | --- | --- | --- |
| Moiety |  | ***^δ^*_C_** | ***^δ^*_H_ (*J* in Hz)** | ***^δ^*_C_** | ***^δ^*_H_ (*J* in Hz)** |
| Kaempferol | **2** | 157.0, qC |  | 158.3, qC |  |
|  | **3** | 133.7, qC |  | 135.5, qC |  |
|  | **4** | 178.8, qC |  | 179.6, qC |  |
|  | **5** | 160.0, qC |  | 161.5, qC |  |
|  | **6** | 98.5, CH | 6.21, *d* (2.0) | 99.9, CH | 6.21, *d* (2.0) |
|  | **7** | 164.2, qC |  | 165.8, qC |  |
|  | **8** | 93.4, CH | 6.41, *d* (2.0) | 94.8, CH | 6.39, *d* (2.0) |
|  | **9** | 154.8, qC |  | 156.4, qC |  |
|  | **10** | 104.3, qC |  | 105.3, qC |  |
|  | **1ˋ** | 121.3, qC |  | 122.4, qC |  |
|  | **2ˋ, 6ˋ** | 131.1, CH | 8.00, *d* (8.0) | 132.3, CH | 8.02, *d* (8.0) |
|  | **3ˋ, 5ˋ** | 114.9, CH | 6.98, *d* (8.0) | 116.4, CH | 6.95, *d* (8.0) |
|  | **4ˋ** | 160.0, qC |  | 162.8, qC |  |
| Glu. | **1ˋˋ** | 104.3, CH | 4.32, *d*, (8.0) | 105.7, CH | 4.54, *d*, (8.0) |
|  | **2 ˋˋ** | 73.4, CH | 3.56, *m* | 80.1, CH | 4.31, *m* |
|  | **3ˋˋ** | 77.7, CH | 3.80, *m* | 78.0, CH | 3.86, *m* |
|  | **4ˋˋ** | 69.9, CH | 3.49, *m* | 71.2, CH | 3.50, *m* |
|  | **5ˋˋ** | 76.8, CH | 3.44, *m* | 77.8, CH | 3.50, *m* |
|  | **6ˋˋ** | 61.2, CH_2_ | 3.78, 3.90, *m* | 62.4, CH_2_ | 3.58, 3.70, *m* |
| Glu. | **1ˋˋˋ** | 103.4, CH | 4.32, *d*, (8.0) |  |  |
|  | **2ˋˋˋ** | 74.1, CH | 3.51, *m* |  |  |
|  | **3ˋˋˋ** | 77.7, CH | 3.86, *m* |  |  |
|  | **4ˋˋˋ** | 69.9, CH | 3.50, *m* |  |  |
|  | **5ˋˋˋ** | 76.5, CH | 3.50, *m* |  |  |
|  | **6ˋˋˋ** | 60.6, CH_2_ | 3.58, 3.70, *m* |  |  |
| Xyl. | **1ˋˋˋ** |  |  | 101.1, CH | 5.43, *d* (8.4) |
|  | **2ˋˋˋ** |  |  | 71.2, CH | 3.73, *m* |
|  | **3ˋˋˋ** |  |  | 75.2, CH | 3.70, *m* |
|  | **4ˋˋˋ** |  |  | 71.0, CH | 3.87, *m* |
|  | **5ˋˋˋ** |  |  | 65.1, CH_2_ | 3.68, 3.39 |

qC, quaternary, CH, methine, CH_2_, methylene carbons, Glc, *β*-D-glucopyranosyl, Xyl., *β-*D-xylopyranosyl.

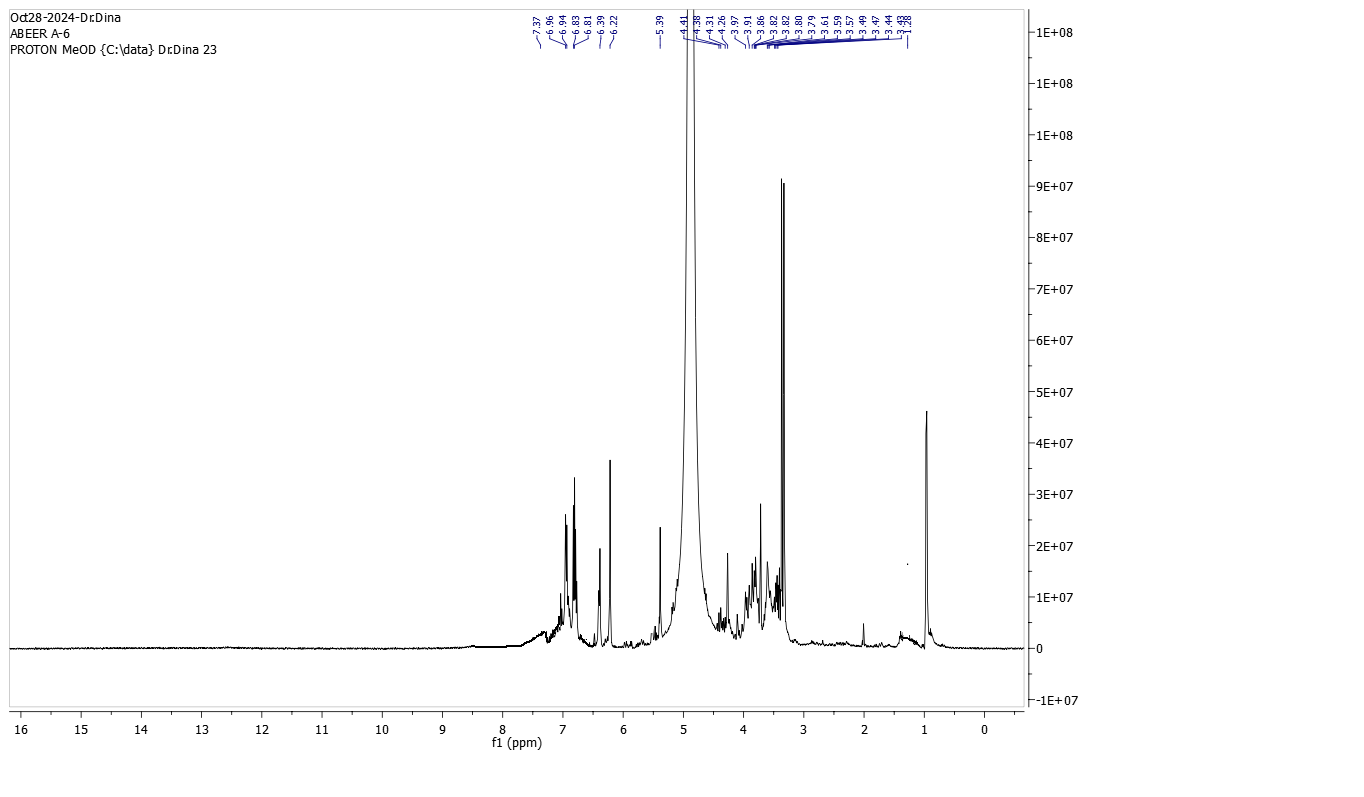


**Figure S5.** ^1^H NMR spectrum of compound **3** (quercetin 3-*O*-rhamnoside) measured in CD_3_OD at 400 MHz.


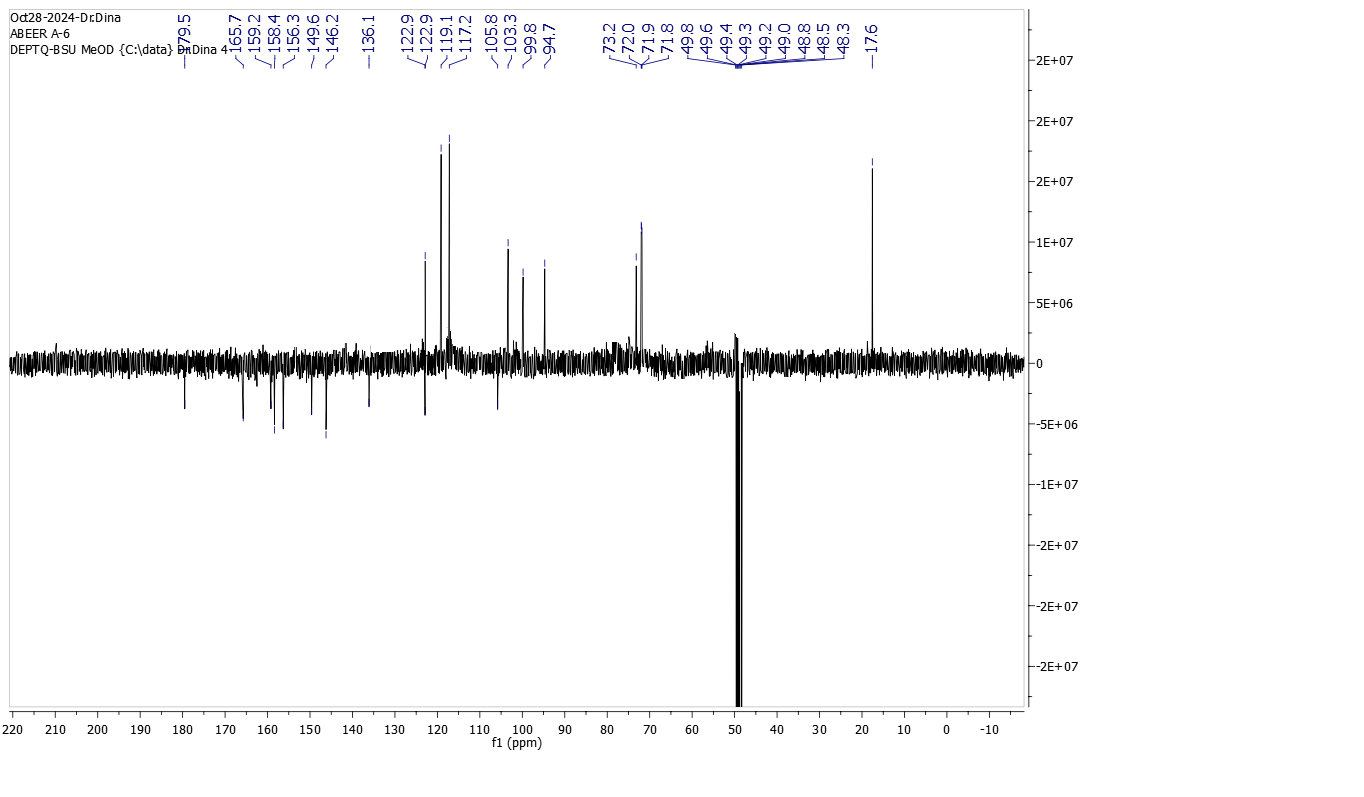


**Figure S6.** DEPT-Q NMR spectrum of compound **3** (quercetin 3-*O*-rhamnoside) measured in CD_3_OD at 100 MHz.

**Table S2.** DEPT-Q (400 MHz) and ^1^H (100 MHz) NMR data for compound **3** in CD_3_OD; carbon multiplicities were determined by DEPT-Q experiments.

|  | Position | 1 | |
| --- | --- | --- | --- |
| Moiety |  | ***^δ^*_C_** | ***^δ^*_H_ (*J* in Hz)** |
| Quercetin | **2** | 158.4, qC |  |
|  | **3** | 136.1, qC |  |
|  | **4** | 179.5, qC |  |
|  | **5** | 159.2, qC |  |
|  | **6** | 99.8, CH | 6.22, *d* (2.0) |
|  | **7** | 165.7, qC |  |
|  | **8** | 94.7, CH | 6.39, *d* (2.0) |
|  | **9** | 156.3, qC |  |
|  | **10** | 105.8, qC |  |
|  | **1ˋ** | 122.9, qC |  |
|  | **2ˋ** | 117.2, CH | 7.37, *d* (2.0) |
|  | **3ˋ** | 149.6, qC |  |
|  | **4ˋ** | 146.2, qC |  |
|  | **5ˋ** | 119.1, CH | 6.83, *d* (8.0) |
|  | **6ˋ** | 122.9, CH | 6.96, *d* (8.0) |
| Rha. | **1ˋˋ** | 103.3, CH | 5.39, *s* |
|  | **2ˋˋ** | 71.9, CH | 3.61, *m* |
|  | **3ˋˋ** | 72.0, CH | 3.60, *m* |
|  | **4ˋˋ** | 73.2, CH | 3.60, *m* |
|  | **5ˋˋ** | 71.8, CH | 4.11, *m* |
|  | **6ˋˋ** | 17.6, CH_3_ | 1.28, *d*, (6.0) |

qC, quaternary, CH, methine, CH_3_, methyl carbons, Rha, *α*-L-rhamnopyranosyl.

**Figure S7.** ^1^H NMR spectrum of compound **4** (procyanidin A2) measured in CD_3_OD at 400 MHz.

**Figure S8.** DEPT-Q NMR spectrum of compound **4** (procyanidin A2) measured in CD_3_OD at 100 MHz.

**Figure S9.** ^1^H NMR spectrum of compound **5** (procyanidin A2 3-*O*-glucoside) measured in CD_3_OD at 400 MHz.

**Figure S10.** DEPT-Q NMR spectrum of compound **5** (procyanidin A2 3-*O*-glucoside) measured in CD_3_OD at 100 MHz.

**Table S3.** DEPT-Q (400 MHz) and ^1^H (100 MHz) NMR data for compounds **4** and **5** in CD_3_OD; carbon multiplicities were determined by DEPT-Q experiments.

|  | Position | 4 | | 5 | |
| --- | --- | --- | --- | --- | --- |
| Moiety |  | ***^δ^*_C_** | ***^δ^*_H_ (*J* in Hz)** | ***^δ^*_C_** | ***^δ^*_H_ (*J* in Hz)** |
| Upper epicatechin unit | **2** | 102.4, qC |  | 102.4, qC |  |
|  | **3** | 66.9, CH | 3.96, *m* | 67.5, CH | 3.96, *m* |
|  | **4** | 29.2, CH | 2.79, *m* | 28.8, CH | 2.79, *m* |
|  | **4a** | 104.3, qC |  | 104.3, qC |  |
|  | **5** | 158.0, qC |  | 158.0, qC |  |
|  | **6** | 98.3, CH | 6.22, *d* (2.0) | 98.3, CH | 6.22, *d* (2.0) |
|  | **7** | 156.9, qC |  | 156.9, qC |  |
|  | **8** | 96.6, CH | 6.39, *d* (2.0) | 96.5, CH | 6.39, *d* (2.0) |
|  | **8a** | 156.4, qC |  | 156.4, qC |  |
|  | **1ˋ** | 131.1, qC |  | 131.1, qC |  |
|  | **2ˋ** | 115.6, CH | 7.37, *d* (2.0) | 115.6, CH | 7.37, *d* (2.0) |
|  | **3ˋ** | 145.8, qC |  | 145.8, qC |  |
|  | **4ˋ** | 146.2, qC |  | 146.2, qC |  |
|  | **5ˋ** | 115.9, CH | 6.83, *d* (8.0) | 115.9, CH | 6.83, *d* (8.0) |
|  | **6ˋ** | 119.8, CH | 6.96, *d* (8.0) | 119.8, CH | 6.96, *d* (8.0) |
| Terminal epicatechin unit | **2** | 81.8, CH | 4.55, *d,* (7.5) | 81.6, CH | 4.55, *d,* (7.5) |
|  | **3** | 68.0, CH | 3.96, *m* | 68.0, CH | 4.25, *m* |
|  | **4** | 29.8, CH_2_ | 2.69, dd, (15.9, 8.4), 2.84, dd, (15.9, 5.4) | 29.8, CH_2_ | 2.49, dd, (15.9, 8.4), 2.64, dd, (15.9, 5.4) |
|  | **4a** | 105.8, qC |  | 105.8, qC |  |
|  | **5** | 158.0, qC |  | 158.0, qC |  |
|  | **6** | 98.3, CH | 6.22, *d* (2.0) | 98.3, CH | 6.22, *d* (2.0) |
|  | **7** | 156.8, qC |  | 156.8, qC |  |
|  | **8** | 107.3, qC |  | 107.3, qC |  |
|  | **8a** | 154.1, qC |  | 154.1, qC |  |
|  | **1ˋ** | 131.4, qC |  | 131.4, qC |  |
|  | **2ˋ** | 115.9, CH | 7.37, *d* (2.0) | 115.9, CH | 7.37, *d* (2.0) |
|  | **3ˋ** | 145.5, qC |  | 145.5, qC |  |
|  | **4ˋ** | 146.6, qC |  | 146.6, qC |  |
|  | **5ˋ** | 116.0, CH | 6.83, *d* (8.0) | 116.0, CH | 6.83, *d* (8.0) |
|  | **6ˋ** | 120.3, CH | 6.96, *d* (8.0) | 120.3, CH | 6.96, *d* (8.0) |
| Glu. | **1ˋˋ** |  |  | 110.4, CH | 4.32, *d*, (8.0) |
|  | **2 ˋˋ** |  |  | 72.5, CH | 3.51, *m* |
|  | **3ˋˋ** |  |  | 80.2, CH | 3.86, *m* |
|  | **4ˋˋ** |  |  | 69.9, CH | 3.50, *m* |
|  | **5ˋˋ** |  |  | 78.8, CH | 3.50, *m* |
|  | **6ˋˋ** |  |  | 62.3, CH_2_ | 3.58, 3.70, *m* |

qC, quaternary, CH, methine, CH_2_, methylene carbons, Glc, *β*-D-glucopyranosyl.

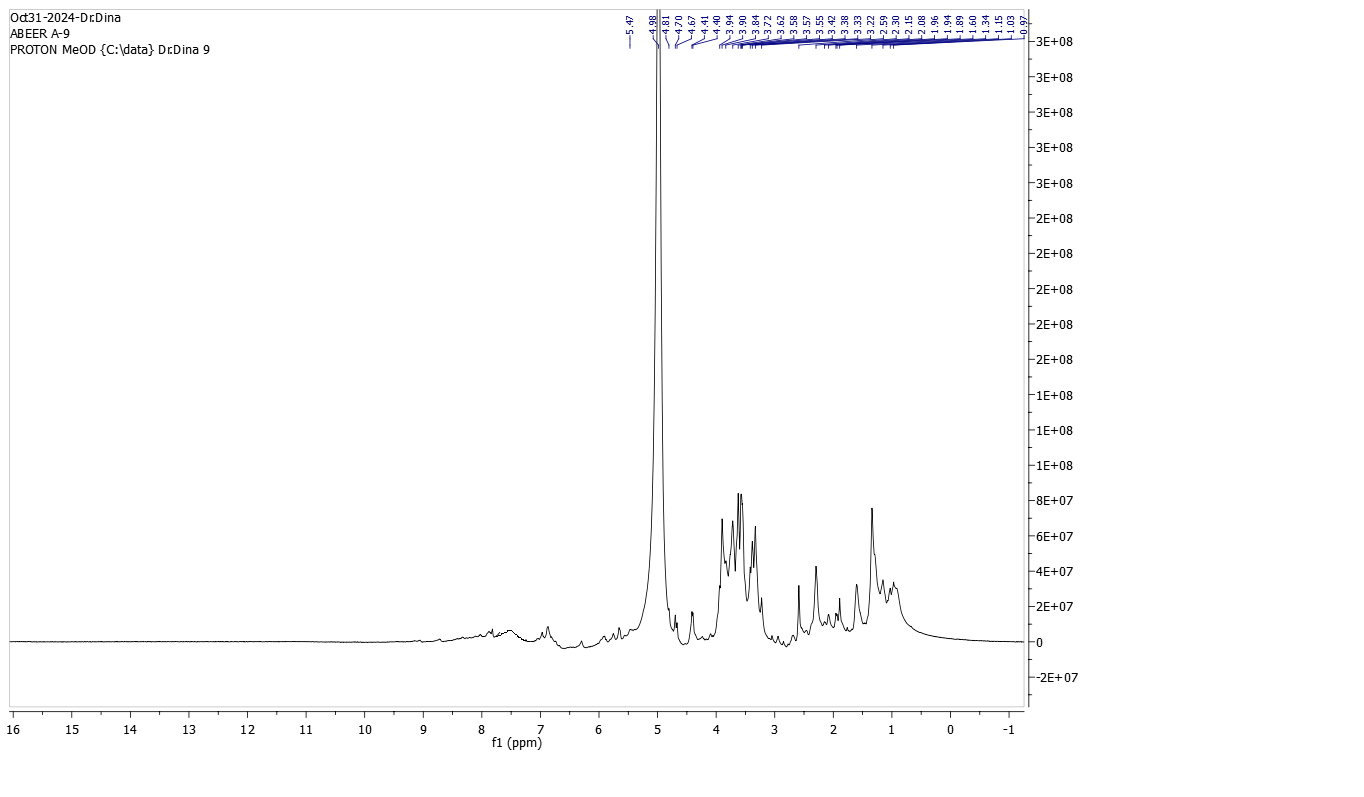


**Figure S11.** ^1^H NMR spectrum of compound **6** ((2*S*)-2-hydroxy-3-[(9*Z*,12*Z*)-1-oxo-9,12-octadecadien-1-yl]oxy]propyl-*O-β*-D-galactopyranoside) measured in CD_3_OD at 400 MHz.

**Table S4.** DEPT-Q (400 MHz) and ^1^H (100 MHz) NMR data for compounds **6** and **8** in CD_3_OD; and CDCl_3_ carbon multiplicities were determined by DEPT-Q experiments.

|  | Position | 6 | | 8 | |
| --- | --- | --- | --- | --- | --- |
| Moiety |  | ***^δ^*_C_** | ***^δ^*_H_ (*J* in Hz)** | ***^δ^*_C_** | ***^δ^*_H_ (*J* in Hz)** |
| Glycerol unit | **1** | 64.4, CH_2_ | 3.89, *m* | 102.4, qC |  |
|  | **2** | 73.7, CH | 3.96, *m* | 67.5, CH | 3.96, *m* |
|  | **3** | 71.7, CH_2_ | 3.79, *m* | 28.8, CH | 2.79, *m* |
| Linoleic acid unit | **1ˋ** | 179.7, qC |  | 179.7, qC |  |
|  | **2ˋ** | 34.9, CH_2_ | 2.35, *m* | 34.9, CH_2_ | 2.35, *m* |
|  | **3ˋ** | 25.9, CH_2_ | 1.51, *m* | 25.9, CH_2_ | 1.51, *m* |
|  | **4ˋ** | 29.9, CH_2_ | 1.31-1.38, *m* | 29.9, CH_2_ | 1.31-1.38, *m* |
|  | **5ˋ** | 29.9, CH_2_ | 1.31-1.38, *m* | 29.9, CH_2_ | 1.31-1.38, *m* |
|  | **6ˋ** | 29.9, CH_2_ | 1.31-1.38, *m* | 29.9, CH_2_ | 1.31-1.38, *m* |
|  | **7ˋ** | 29.9, CH_2_ | 1.31-1.38, *m* | 29.9, CH_2_ | 1.31-1.38, *m* |
|  | **8ˋ** | 29.8, CH_2_ | 2.04-2.09, *m* | 29.8, CH_2_ | 2.04-2.09, *m* |
|  | **9ˋ** | 129.2, CH | 5.02-5.31, *m* | 129.2, CH | 5.02-5.31, *m* |
|  | **10ˋ** | 128.6, CH | 5.02-5.31, *m* | 128.6, CH | 5.02-5.31, *m* |
|  | **11ˋ** | 25.9, CH_2_ | 2.78, *m* | 25.9, CH_2_ | 2.78, *m* |
|  | **12ˋ** | 129.1, CH | 5.02-5.31, *m* | 129.1, CH | 5.02-5.31, *m* |
|  | **13ˋ** | 129.2, CH | 5.02-5.31, *m* | 129.2, CH | 5.02-5.31, *m* |
|  | **14ˋ** | 29.8, CH_2_ | 2.04-2.09, *m* | 29.8, CH_2_ | 2.04-2.09, *m* |
|  | **15ˋ** | 30.0, CH_2_ | 1.31-1.38, *m* | 30.0, CH_2_ | 1.31-1.38, *m* |
|  | **16ˋ** | 25.9, CH_2_ | 1.31-1.38, *m* | 25.9, CH_2_ | 1.31-1.38, *m* |
|  | **17ˋ** | 34.9, CH_2_ | 1.31-1.38, *m* | 34.9, CH_2_ | 1.31-1.38, *m* |
|  | **18ˋ** | 12.4, CH_3_ | 0.90, *t* | 12.4, CH_3_ | 0.90, *t* |
| Glu. | **1ˋˋ** | 103.1, CH | 4.32, *d*, (8.0) |  |  |
|  | **2 ˋˋ** | 75.0, CH | 3.51, *m* |  |  |
|  | **3ˋˋ** | 77.7, CH | 3.86, *m* |  |  |
|  | **4ˋˋ** | 71.5, CH | 3.50, *m* |  |  |
|  | **5ˋˋ** | 77.8, CH | 3.50, *m* |  |  |
|  | **6ˋˋ** | 62.6, CH_2_ | 3.58, 3.70, *m* |  |  |

CH, methine, CH_2_, methylene, CH_3_, methyl carbons, Glc, *β*-D-glucopyranosyl.


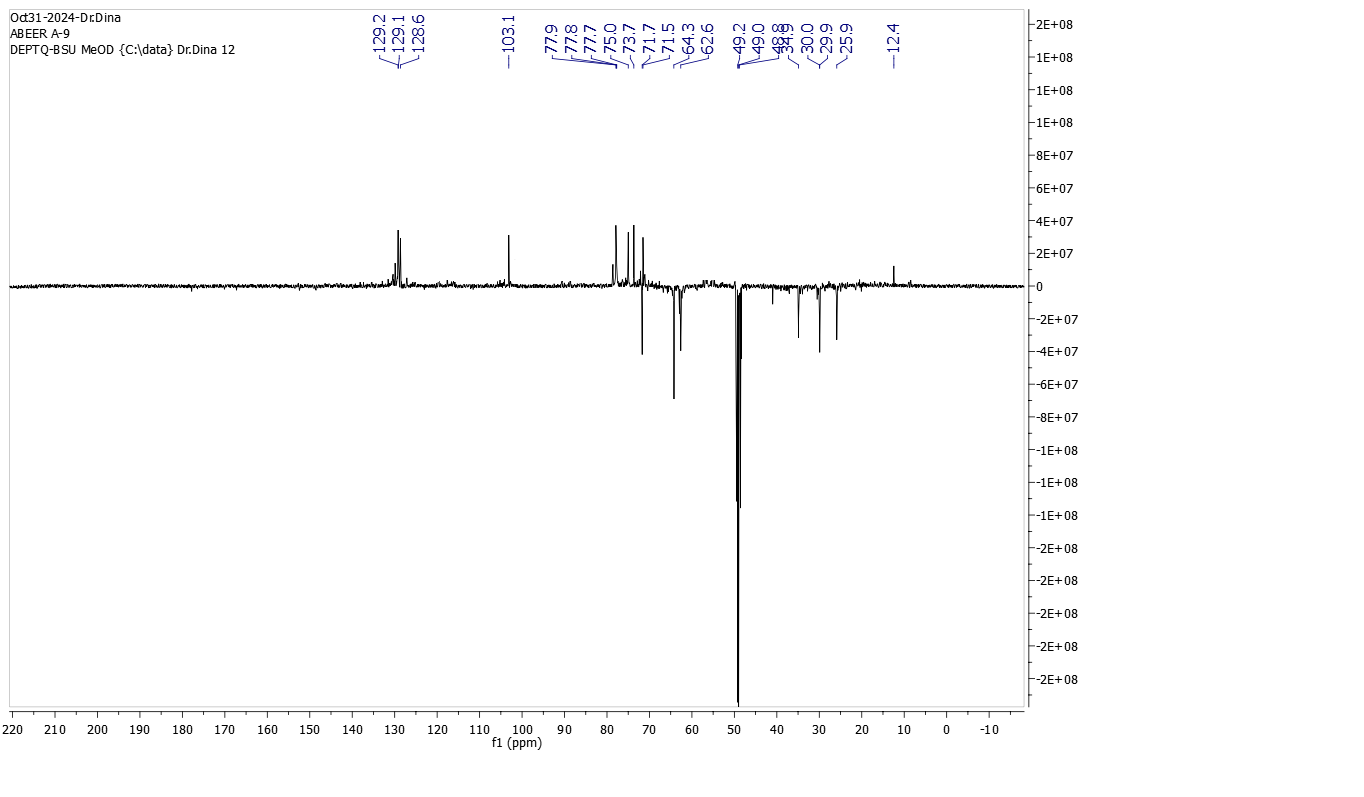


**Figure S12.** DEPT-Q NMR spectrum of compound **6** ((2*S*)-2-hydroxy-3-[(9*Z*,12*Z*)-1-oxo-9,12-octadecadien-1-yl]oxy]propyl-*O-β*-D-galactopyranoside) measured in CD_3_OD at 100 MHz.

**Figure S13.** ^1^H NMR spectrum of compound **7** (palmitic acid) measured in CDCl_3_ at 400 MHz.

**Figure S14.** ^1^H NMR spectrum of compound **8** (linoleic acid) measured in CDCl_3_ at 400 MHz.

**Table S5:** List of proteins related to MRSA.

| **Protein** | **Online library source** |
| --- | --- |
| **IL10RA** - Interleukin-10 Receptor Subunit Alpha | **pharmGKB** |
| **IL1B** - Interleukin-1 Beta | **pharmGKB** |
| **IL10RB** - Interleukin-10 Receptor Subunit Beta | **pharmGKB** |
| **IL4R** - Interleukin-4 Receptor | **pharmGKB** |
| **SOCS3** - Suppressor of Cytokine Signaling 3 | **pharmGKB** |
| **CNTF** - Ciliary Neurotrophic Factor | **pharmGKB** |
| **IL12RB1** - Interleukin-12 Receptor Subunit Beta-1 | **pharmGKB** |
| **IL9** - Interleukin-9 | **pharmGKB** |
| **IL2RA** - Interleukin-2 Receptor Subunit Alpha | **pharmGKB** |
| **IL11** - Interleukin-11 | **pharmGKB** |
| **EGFR** - Epidermal Growth Factor Receptor | **pharmGKB** |
| **MYD88** - Myeloid Differentiation Primary Response 88 | **pharmGKB** |
| **REG3A** - Regenerating Islet-Derived Protein 3 Alpha | **pharmGKB** |
| **IL28A** - Interleukin-28A (IFN-λ2) | **pharmGKB** |
| **JAK2** - Janus Kinase 2 | **pharmGKB** |
| **myo-inositol** - Myo-inositol, a sugar-like molecule involved in signaling | **pharmGKB** |
| **IRAK2** - Interleukin-1 Receptor-Associated Kinase 2 | **pharmGKB** |
| **IL12RB2** - Interleukin-12 Receptor Subunit Beta-2 | **pharmGKB** |
| **IFNG** - Interferon-Gamma | **pharmGKB** |
| **IL21** - Interleukin-21 | **pharmGKB** |
| **IL29** - Interleukin-29 (IFN-λ1) | **pharmGKB** |
| **IL17A** - Interleukin-17A | **pharmGKB** |
| **IL17RA** - Interleukin-17 Receptor A | **NCBI** |
| **STAT3** - Signal Transducer and Activator of Transcription 3 | **NCBI** |
| **TSLP** - Thymic Stromal Lymphopoietin | **NCBI** |
| **IL6R** - Interleukin-6 Receptor | **NCBI** |
| **SOCS1** - Suppressor of Cytokine Signaling 1 | **NCBI** |
| **GAPDH** - Glyceraldehyde-3-Phosphate Dehydrogenase | **NCBI** |
| **IL4** - Interleukin-4 | **NCBI** |
| **IL10** - Interleukin-10 | **NCBI** |
| **SRC** - SRC Proto-Oncogene, Non-Receptor Tyrosine Kinase | **NCBI** |
| **IFNGR1** - Interferon-Gamma Receptor 1 | **NCBI** |
| **IL11RA** - Interleukin-11 Receptor Subunit Alpha | **NCBI** |
| **IL1R1** - Interleukin-1 Receptor Type 1 | **NCBI** |
| **IL6ST** - Interleukin-6 Signal Transducer (gp130) | **NCBI** |
| **STAT5B** - Signal Transducer and Activator of Transcription 5B | **NCBI** |
| **STAT5A** - Signal Transducer and Activator of Transcription 5A | **NCBI** |
| **IL6** - Interleukin-6 | **NCBI** |
| **IL13** - Interleukin-13 | **NCBI** |
| **STAT6** - Signal Transducer and Activator of Transcription 6 | **NCBI** |
| **IL23R** - Interleukin-23 Receptor | **NCBI** |
| **HPRT1** - Hypoxanthine Phosphoribosyltransferase 1 | **NCBI** |
| **IL27RA** - Interleukin-27 Receptor Subunit Alpha | **NCBI** |
| **IRAK4** - Interleukin-1 Receptor-Associated Kinase 4 | **NCBI** |
| **IL22** - Interleukin-22 | **NCBI** |
| **IL17F** - Interleukin-17F | **NCBI** |
| **TRAF6** - TNF Receptor-Associated Factor 6 | **NCBI** |
| **IL23A** - Interleukin-23 Subunit Alpha | **NCBI** |
| **STAT1** - Signal Transducer and Activator of Transcription 1 | **NCBI** |
| **IL12B** - Interleukin-12 Subunit Beta | **NCBI** |
| **CSF3** - Colony Stimulating Factor 3 (Granulocyte Colony-Stimulating Factor) | **NCBI** |

**PharmGKB (Pharmacogenomics Knowledgebase [www.pharmgkb.org](http://www.pharmgkb.org/))**

**NCBI (National Center for Biotechnology Information [www.ncbi.nlm.nih.gov](https://www.ncbi.nlm.nih.gov/))**

**Table S6:** GO enrichment entry.

| **Category** | **Description** |
| --- | --- |
| GO:0019221 | Cytokine-mediated signaling pathway |
| GO:0071345 | Cellular response to cytokine stimulus |
| GO:0001819 | Positive regulation of cytokine production |
| GO:0042531 | Positive regulation of tyrosine phosphorylation of STAT protein |
| GO:0006955 | Immune response |
| GO:0002376 | Immune system process |
| GO:0010628 | Positive regulation of gene expression |
| GO:0050730 | Regulation of peptidyl-tyrosine phosphorylation |
| GO:0050731 | Positive regulation of peptidyl-tyrosine phosphorylation |
| GO:0001817 | Regulation of cytokine production |
| GO:0051240 | Positive regulation of multicellular organismal process |
| GO:0006952 | Defense response |
| GO:0008284 | Positive regulation of cell population proliferation |
| GO:0006954 | Inflammatory response |
| GO:0007166 | Cell surface receptor signaling pathway |
| GO:0051239 | Regulation of multicellular organismal process |
| GO:0042127 | Regulation of cell population proliferation |
| GO:0007165 | Signal transduction |
| GO:0071310 | Cellular response to organic substance |
| GO:0010033 | Response to organic substance |
| GO:1904892 | Regulation of receptor signaling pathway via STAT |
| GO:1904894 | Positive regulation of receptor signaling pathway via STAT |
| GO:0002682 | Regulation of immune system process |
| GO:0048584 | Positive regulation of response to stimulus |
| GO:0051707 | Response to other organism |
| GO:0046425 | Regulation of receptor signaling pathway via JAK-STAT |
| GO:0001775 | Cell activation |
| GO:0002684 | Positive regulation of immune system process |
| GO:0046427 | Positive regulation of receptor signaling pathway via JAK-STAT |
| GO:0009893 | Positive regulation of metabolic process |
| GO:0048518 | Positive regulation of biological process |
| GO:0045321 | Leukocyte activation |
| GO:0051716 | Cellular response to stimulus |
| GO:0010604 | Positive regulation of macromolecule metabolic process |
| GO:0050896 | Response to stimulus |
| GO:0098542 | Defense response to other organism |
| GO:0048583 | Regulation of response to stimulus |
| GO:0070665 | Positive regulation of leukocyte proliferation |
| GO:0050865 | Regulation of cell activation |
| GO:0001934 | Positive regulation of protein phosphorylation |
| GO:0002696 | Positive regulation of leukocyte activation |
| GO:0002694 | Regulation of leukocyte activation |
| GO:0050671 | Positive regulation of lymphocyte proliferation |
| GO:0006950 | Response to stress |
| GO:0051249 | Regulation of lymphocyte activation |
| GO:0002697 | Regulation of immune effector process |
| GO:0070663 | Regulation of leukocyte proliferation |
| GO:0051251 | Positive regulation of lymphocyte activation |
| GO:0001932 | Regulation of protein phosphorylation |
| GO:0048522 | Positive regulation of cellular process |
| GO:1903706 | Regulation of hemopoiesis |
| GO:0042325 | Regulation of phosphorylation |
| GO:0032660 | Regulation of interleukin-17 production |
| GO:1903037 | Regulation of leukocyte cell-cell adhesion |
| GO:0009605 | Response to external stimulus |
| GO:0051247 | Positive regulation of protein metabolic process |
| GO:0031347 | Regulation of defense response |
| GO:0022407 | Regulation of cell-cell adhesion |
| GO:0050778 | Positive regulation of immune response |
| GO:0032101 | Regulation of response to external stimulus |
| GO:0051094 | Positive regulation of developmental process |
| GO:0050863 | Regulation of T cell activation |
| GO:1902105 | Regulation of leukocyte differentiation |
| GO:0002699 | Positive regulation of immune effector process |
| GO:0022409 | Positive regulation of cell-cell adhesion |
| GO:0002521 | Leukocyte differentiation |
| GO:1903039 | Positive regulation of leukocyte cell-cell adhesion |
| GO:0050776 | Regulation of immune response |
| GO:1902107 | Positive regulation of leukocyte differentiation |
| GO:0045595 | Regulation of cell differentiation |
| GO:0009617 | Response to bacterium |
| GO:0042102 | Positive regulation of T cell proliferation |
| GO:0045597 | Positive regulation of cell differentiation |
| GO:0002819 | Regulation of adaptive immune response |
| GO:0030155 | Regulation of cell adhesion |
| GO:0050727 | Regulation of inflammatory response |
| GO:0050870 | Positive regulation of T cell activation |
| GO:0002821 | Positive regulation of adaptive immune response |
| GO:2000026 | Regulation of multicellular organismal development |
| GO:0009967 | Positive regulation of signal transduction |
| GO:0050793 | Regulation of developmental process |
| GO:0030097 | Hemopoiesis |
| GO:0045785 | Positive regulation of cell adhesion |
| GO:1900017 | Positive regulation of cytokine production involved in inflammatory response |
| GO:0032755 | Positive regulation of interleukin-6 production |
| GO:0002822 | Regulation of adaptive immune response based on somatic recombination of immune receptors built from immunoglobulin superfamily domains |
| GO:0032819 | Positive regulation of natural killer cell proliferation |
| GO:0032722 | Positive regulation of chemokine production |
| GO:0032740 | Positive regulation of interleukin-17 production |
| GO:0010468 | Regulation of gene expression |
| GO:0051241 | Negative regulation of multicellular organismal process |
| GO:0032103 | Positive regulation of response to external stimulus |
| GO:0002824 | Positive regulation of adaptive immune response based on somatic recombination of immune receptors built from immunoglobulin superfamily domains |
| GO:0032675 | Regulation of interleukin-6 production |
| GO:0046649 | Lymphocyte activation |
| GO:0032729 | Positive regulation of interferon-gamma production |
| GO:0007259 | Receptor signaling pathway via JAK-STAT |
| GO:0019222 | Regulation of metabolic process |
| GO:0051246 | Regulation of protein metabolic process |
| GO:0080134 | Regulation of response to stress |
| GO:0045580 | Regulation of T cell differentiation |
| GO:0002250 | Adaptive immune response |
| GO:0050794 | Regulation of cellular process |
| GO:0031325 | Positive regulation of cellular metabolic process |
| GO:0002703 | Regulation of leukocyte mediated immunity |
| GO:0045639 | Positive regulation of myeloid cell differentiation |
| GO:0051142 | Positive regulation of NK T cell proliferation |
| GO:0002700 | Regulation of production of molecular mediator of immune response |
| GO:0009966 | Regulation of signal transduction |
| GO:0060255 | Regulation of macromolecule metabolic process |
| GO:0046634 | Regulation of alpha-beta T cell activation |
| GO:0051173 | Positive regulation of nitrogen compound metabolic process |
| GO:1903555 | Regulation of tumor necrosis factor superfamily cytokine production |
| GO:0009615 | Response to virus |
| GO:0002706 | Regulation of lymphocyte mediated immunity |
| GO:0031349 | Positive regulation of defense response |
| GO:0002763 | Positive regulation of myeloid leukocyte differentiation |
| GO:0042110 | T cell activation |
| GO:0042742 | Defense response to bacterium |
| GO:0032496 | Response to lipopolysaccharide |
| GO:1903131 | Mononuclear cell differentiation |
| GO:0050729 | Positive regulation of inflammatory response |
| GO:1903557 | Positive regulation of tumor necrosis factor superfamily cytokine production |
| GO:0046635 | Positive regulation of alpha-beta T cell activation |
| GO:0045637 | Regulation of myeloid cell differentiation |
| GO:0030098 | Lymphocyte differentiation |
| GO:0001818 | Negative regulation of cytokine production |
| GO:0002708 | Positive regulation of lymphocyte mediated immunity |
| GO:0032680 | Regulation of tumor necrosis factor production |
| GO:0002460 | Adaptive immune response based on somatic recombination of immune receptors built from immunoglobulin superfamily domains |
| GO:0045672 | Positive regulation of osteoclast differentiation |
| GO:0046640 | Regulation of alpha-beta T cell proliferation |
| GO:0002761 | Regulation of myeloid leukocyte differentiation |
| GO:0045087 | Innate immune response |
| GO:0002252 | Immune effector process |
| GO:0048519 | Negative regulation of biological process |
| GO:0002709 | Regulation of T cell mediated immunity |
| GO:0045621 | Positive regulation of lymphocyte differentiation |
| GO:0098586 | Cellular response to virus |
| GO:2000316 | Regulation of T-helper 17 type immune response |
| GO:0002711 | Positive regulation of T cell mediated immunity |
| GO:0002702 | Positive regulation of production of molecular mediator of immune response |
| GO:0002825 | Regulation of T-helper 1 type immune response |
| GO:0002366 | Leukocyte activation involved in immune response |
| GO:0002828 | Regulation of type 2 immune response |
| GO:0032653 | Regulation of interleukin-10 production |
| GO:0048660 | Regulation of smooth muscle cell proliferation |
| GO:0046631 | Alpha-beta T cell activation |
| GO:0050829 | Defense response to Gram-negative bacterium |
| GO:0002269 | Leukocyte activation involved in inflammatory response |
| GO:0032760 | Positive regulation of tumor necrosis factor production |
| GO:0002274 | Myeloid leukocyte activation |
| GO:0042093 | T-helper cell differentiation |
| GO:0061900 | Glial cell activation |
| GO:0045670 | Regulation of osteoclast differentiation |
| GO:0032733 | Positive regulation of interleukin-10 production |
| GO:0002827 | Positive regulation of T-helper 1 type immune response |
| GO:0031323 | Regulation of cellular metabolic process |
| GO:0045582 | Positive regulation of T cell differentiation |
| GO:0002286 | T cell activation involved in immune response |
| GO:0002637 | Regulation of immunoglobulin production |
| GO:0032747 | Positive regulation of interleukin-23 production |
| GO:0030217 | T cell differentiation |
| GO:2000514 | Regulation of CD4-positive, alpha-beta T cell activation |
| GO:0046651 | Lymphocyte proliferation |
| GO:0002360 | T cell lineage commitment |
| GO:0033993 | Response to lipid |
| GO:0002285 | Lymphocyte activation involved in immune response |
| GO:0008283 | Cell population proliferation |
| GO:0043370 | Regulation of CD4-positive, alpha-beta T cell differentiation |
| GO:0042104 | Positive regulation of activated T cell proliferation |
| GO:0010629 | Negative regulation of gene expression |
| GO:0043380 | Regulation of memory T cell differentiation |
| GO:0001774 | Microglial cell activation |
| GO:0032731 | Positive regulation of interleukin-1 beta production |
| GO:0031341 | Regulation of cell killing |
| GO:0042116 | Macrophage activation |
| GO:0043372 | Positive regulation of CD4-positive, alpha-beta T cell differentiation |
| GO:0070102 | interleukin-6-mediated signaling pathway |
| GO:0033138 | Positive regulation of peptidyl-serine phosphorylation |
| GO:0002685 | Regulation of leukocyte migration |
| GO:2000318 | Positive regulation of T-helper 17 type immune response |
| GO:0008285 | Negative regulation of cell population proliferation |
| GO:0051171 | Regulation of nitrogen compound metabolic process |
| GO:2000330 | Positive regulation of T-helper 17 cell lineage commitment |
| GO:0048523 | Negative regulation of cellular process |
| GO:0051607 | Defense response to virus |
| GO:0045622 | Regulation of T-helper cell differentiation |
| GO:0032645 | Regulation of granulocyte macrophage colony-stimulating factor production |
| GO:0034341 | Response to interferon-gamma |
| GO:0030335 | Positive regulation of cell migration |
| GO:0002683 | Negative regulation of immune system process |
| GO:0050830 | Defense response to Gram-positive bacterium |
| GO:0030099 | Myeloid cell differentiation |
| GO:0080090 | Regulation of primary metabolic process |
| GO:0050691 | Regulation of defense response to virus by host |
| GO:0032674 | Regulation of interleukin-5 production |
| GO:2000319 | Regulation of T-helper 17 cell differentiation |
| GO:0071222 | Cellular response to lipopolysaccharide |
| GO:0002573 | Myeloid leukocyte differentiation |
| GO:0032677 | Regulation of interleukin-8 production |
| GO:0032656 | Regulation of interleukin-13 production |
| GO:0030334 | Regulation of cell migration |
| GO:2000338 | Regulation of chemokine (C-X-C motif) ligand 1 production |
| GO:0002673 | Regulation of acute inflammatory response |
| GO:1903530 | Regulation of secretion by cell |
| GO:0045624 | Positive regulation of T-helper cell differentiation |
| GO:0002639 | Positive regulation of immunoglobulin production |
| GO:0010536 | Positive regulation of activation of Janus kinase activity |
| GO:0002687 | Positive regulation of leukocyte migration |
| GO:0048585 | Negative regulation of response to stimulus |
| GO:0001916 | Positive regulation of T cell mediated cytotoxicity |
| GO:0071346 | Cellular response to interferon-gamma |
| GO:0043382 | Positive regulation of memory T cell differentiation |
| GO:0070120 | Ciliary neurotrophic factor-mediated signaling pathway |
| GO:0031343 | Positive regulation of cell killing |
| GO:0097191 | Extrinsic apoptotic signaling pathway |
| GO:0002230 | Positive regulation of defense response to virus by host |
| GO:1901700 | Response to oxygen-containing compound |
| GO:0050777 | Negative regulation of immune response |
| GO:0002295 | T-helper cell lineage commitment |
| GO:1901731 | Positive regulation of platelet aggregation |
| GO:0009620 | Response to fungus |
| GO:0042113 | B cell activation |
| GO:0006959 | Humoral immune response |
| GO:0002831 | Regulation of response to biotic stimulus |
| GO:0007260 | Tyrosine phosphorylation of STAT protein |
| GO:0097398 | Cellular response to interleukin-17 |
| GO:0002829 | Negative regulation of type 2 immune response |
| GO:0072538 | T-helper 17 type immune response |
| GO:0002526 | Acute inflammatory response |
| GO:1902533 | Positive regulation of intracellular signal transduction |
| GO:0032700 | Negative regulation of interleukin-17 production |
| GO:0032725 | Positive regulation of granulocyte macrophage colony-stimulating factor production |
| GO:0006953 | Acute-phase response |
| GO:0050864 | Regulation of B cell activation |
| GO:0002443 | Leukocyte mediated immunity |
| GO:0032735 | Positive regulation of interleukin-12 production |
| GO:0045058 | T cell selection |
| GO:0048661 | Positive regulation of smooth muscle cell proliferation |
| GO:0060548 | Negative regulation of cell death |
| GO:0031328 | Positive regulation of cellular biosynthetic process |
| GO:0048143 | Astrocyte activation |
| GO:0043066 | Negative regulation of apoptotic process |
| GO:0006915 | Apoptotic process |
| GO:0045581 | Negative regulation of T cell differentiation |
| GO:0050871 | Positive regulation of B cell activation |
| GO:0009987 | Cellular process |
| GO:1900221 | Regulation of amyloid-beta clearance |
| GO:0051093 | Negative regulation of developmental process |
| GO:0010557 | Positive regulation of macromolecule biosynthetic process |
| GO:0002690 | Positive regulation of leukocyte chemotaxis |
| GO:0046883 | Regulation of hormone secretion |
| GO:0051048 | Negative regulation of secretion |
| GO:0033005 | Positive regulation of mast cell activation |
| GO:0045596 | Negative regulation of cell differentiation |
| GO:0050708 | Regulation of protein secretion |
| GO:0048513 | Animal organ development |
| GO:0018108 | Peptidyl-tyrosine phosphorylation |
| GO:0042981 | Regulation of apoptotic process |
| GO:2000340 | Positive regulation of chemokine (C-X-C motif) ligand 1 production |
| GO:1903707 | Negative regulation of hemopoiesis |
| GO:0010941 | Regulation of cell death |
| GO:0048708 | Astrocyte differentiation |
| GO:0050832 | Defense response to fungus |
| GO:0051091 | Positive regulation of DNA-binding transcription factor activity |
| GO:0002675 | Positive regulation of acute inflammatory response |
| GO:0043410 | Positive regulation of MAPK cascade |
| GO:0002718 | Regulation of cytokine production involved in immune response |
| GO:0032757 | Positive regulation of interleukin-8 production |
| GO:0002698 | Negative regulation of immune effector process |
| GO:0022408 | Negative regulation of cell-cell adhesion |
| GO:0030225 | Macrophage differentiation |
| GO:0010573 | Vascular endothelial growth factor production |
| GO:0038165 | Oncostatin-M-mediated signaling pathway |
| GO:0038196 | Type III interferon signaling pathway |
| GO:0071396 | Cellular response to lipid |
| GO:0072540 | T-helper 17 cell lineage commitment |
| GO:0030183 | B cell differentiation |
| GO:0050868 | Negative regulation of T cell activation |
| GO:0043467 | Regulation of generation of precursor metabolites and energy |
| GO:0097529 | Myeloid leukocyte migration |
| GO:0045860 | Positive regulation of protein kinase activity |
| GO:0014068 | Positive regulation of phosphatidylinositol 3-kinase signaling |
| GO:0048861 | Leukemia inhibitory factor signaling pathway |
| GO:0097400 | interleukin-17-mediated signaling pathway |
| GO:1900222 | Negative regulation of amyloid-beta clearance |
| GO:1903531 | Negative regulation of secretion by cell |
| GO:0120161 | Regulation of cold-induced thermogenesis |
| GO:0060397 | Growth hormone receptor signaling pathway via JAK-STAT |
| GO:1901214 | Regulation of neuron death |
| GO:0002724 | Regulation of T cell cytokine production |
| GO:0030856 | Regulation of epithelial cell differentiation |
| GO:2001234 | Negative regulation of apoptotic signaling pathway |
| GO:0045935 | Positive regulation of nucleobase-containing compound metabolic process |
| GO:0032754 | Positive regulation of interleukin-5 production |
| GO:0034105 | Positive regulation of tissue remodeling |
| GO:0071803 | Positive regulation of podosome assembly |
| GO:0002251 | Organ or tissue specific immune response |
| GO:0048731 | System development |
| GO:0002449 | Lymphocyte mediated immunity |
| GO:0010648 | Negative regulation of cell communication |
| GO:0045893 | Positive regulation of transcription, DNA-templated |
| GO:0034393 | Positive regulation of smooth muscle cell apoptotic process |
| GO:0060333 | Interferon-gamma-mediated signaling pathway |
| GO:0023057 | Negative regulation of signaling |
| GO:0050900 | Leukocyte migration |
| GO:0016064 | Immunoglobulin mediated immune response |
| GO:0009892 | Negative regulation of metabolic process |
| GO:2000551 | Regulation of T-helper 2 cell cytokine production |
| GO:0030890 | Positive regulation of B cell proliferation |
| GO:0120162 | Positive regulation of cold-induced thermogenesis |
| GO:2001237 | Negative regulation of extrinsic apoptotic signaling pathway |
| GO:0032736 | Positive regulation of interleukin-13 production |
| GO:1901701 | Cellular response to oxygen-containing compound |
| GO:1901857 | Positive regulation of cellular respiration |
| GO:1902622 | Regulation of neutrophil migration |
| GO:2001233 | Regulation of apoptotic signaling pathway |
| GO:0043457 | Regulation of cellular respiration |
| GO:0030154 | Cell differentiation |
| GO:0043306 | Positive regulation of mast cell degranulation |
| GO:0045348 | Positive regulation of MHC class II biosynthetic process |
| GO:0045944 | Positive regulation of transcription by RNA polymerase II |
| GO:1904035 | Regulation of epithelial cell apoptotic process |
| GO:1903799 | Negative regulation of miRNA maturation |
| GO:0042100 | B cell proliferation |
| GO:0048662 | Negative regulation of smooth muscle cell proliferation |
| GO:0051897 | Positive regulation of protein kinase B signaling |
| GO:0002830 | Positive regulation of type 2 immune response |
| GO:0031334 | Positive regulation of protein-containing complex assembly |
| GO:0002823 | Negative regulation of adaptive immune response based on somatic recombination of immune receptors built from immunoglobulin superfamily domains |
| GO:0043030 | Regulation of macrophage activation |
| GO:0010605 | Negative regulation of macromolecule metabolic process |
| GO:0032693 | Negative regulation of interleukin-10 production |
| GO:0033141 | Positive regulation of peptidyl-serine phosphorylation of STAT protein |
| GO:1902004 | Positive regulation of amyloid-beta formation |
| GO:0002384 | Hepatic immune response |
| GO:0002889 | Regulation of immunoglobulin mediated immune response |
| GO:0060557 | Positive regulation of vitamin D biosynthetic process |
| GO:0002922 | Positive regulation of humoral immune response |
| GO:0030101 | Natural killer cell activation |
| GO:0042088 | T-helper 1 type immune response |
| GO:0014015 | Positive regulation of gliogenesis |
| GO:0010660 | Regulation of muscle cell apoptotic process |
| GO:1901215 | Negative regulation of neuron death |
| GO:0051130 | Positive regulation of cellular component organization |
| GO:1903578 | Regulation of ATP metabolic process |
| GO:0002446 | Neutrophil mediated immunity |
| GO:0042098 | T cell proliferation |
| GO:0048856 | Anatomical structure development |
| GO:0002726 | Positive regulation of T cell cytokine production |
| GO:0033861 | Negative regulation of NAD(P)H oxidase activity |
| GO:0038155 | interleukin-23-mediated signaling pathway |
| GO:0060559 | Positive regulation of calcidiol 1-monooxygenase activity |
| GO:1903660 | Negative regulation of complement-dependent cytotoxicity |
| GO:2000635 | Negative regulation of primary miRNA processing |
| GO:0009968 | Negative regulation of signal transduction |
| GO:0034103 | Regulation of tissue remodeling |
| GO:0042832 | Defense response to protozoan |
| GO:0050728 | Negative regulation of inflammatory response |
| GO:0030595 | Leukocyte chemotaxis |
| GO:0071624 | Positive regulation of granulocyte chemotaxis |
| GO:0001959 | Regulation of cytokine-mediated signaling pathway |
| GO:0042119 | Neutrophil activation |
| GO:0048710 | Regulation of astrocyte differentiation |
| GO:0002720 | Positive regulation of cytokine production involved in immune response |
| GO:0035722 | interleukin-12-mediated signaling pathway |
| GO:0035771 | interleukin-4-mediated signaling pathway |
| GO:0038157 | Granulocyte-macrophage colony-stimulating factor signaling pathway |
| GO:0048295 | Positive regulation of isotype switching to IgE isotypes |
| GO:0071353 | Cellular response to interleukin-4 |
| GO:0016310 | Phosphorylation |
| GO:0045921 | Positive regulation of exocytosis |
| GO:0032770 | Positive regulation of monooxygenase activity |
| GO:0150077 | Regulation of neuroinflammatory response |
| GO:1900182 | Positive regulation of protein localization to nucleus |
| GO:1902624 | Positive regulation of neutrophil migration |
| GO:1903580 | Positive regulation of ATP metabolic process |
| GO:2001240 | Negative regulation of extrinsic apoptotic signaling pathway in absence of ligand |
| GO:0002860 | Positive regulation of natural killer cell mediated cytotoxicity directed against tumor cell target |
| GO:0042976 | Activation of Janus kinase activity |
| GO:0072537 | Fibroblast activation |
| GO:1903532 | Positive regulation of secretion by cell |
| GO:0045191 | Regulation of isotype switching |
| GO:0071347 | Cellular response to interleukin-1 |
| GO:0045765 | Regulation of angiogenesis |
| GO:0044093 | Positive regulation of molecular function |
| GO:0090276 | Regulation of peptide hormone secretion |
| GO:0097530 | Granulocyte migration |
| GO:0051341 | Regulation of oxidoreductase activity |
| GO:0002225 | Positive regulation of antimicrobial peptide production |
| GO:0032696 | Negative regulation of interleukin-13 production |
| GO:0045064 | T-helper 2 cell differentiation |
| GO:1903588 | Negative regulation of blood vessel endothelial cell proliferation involved in sprouting angiogenesis |
| GO:0002891 | Positive regulation of immunoglobulin mediated immune response |
| GO:0010720 | Positive regulation of cell development |
| GO:0001937 | Negative regulation of endothelial cell proliferation |
| GO:0045429 | Positive regulation of nitric oxide biosynthetic process |
| GO:0032714 | Negative regulation of interleukin-5 production |
| GO:2000659 | Regulation of interleukin-1-mediated signaling pathway |
| GO:0009889 | Regulation of biosynthetic process |
| GO:0006468 | Protein phosphorylation |
| GO:0014075 | Response to amine |
| GO:0061844 | Antimicrobial humoral immune response mediated by antimicrobial peptide |
| GO:0006110 | Regulation of glycolytic process |
| GO:2000553 | Positive regulation of T-helper 2 cell cytokine production |
| GO:0030857 | Negative regulation of epithelial cell differentiation |
| GO:0009725 | Response to hormone |
| GO:0043523 | Regulation of neuron apoptotic process |
| GO:0002676 | Regulation of chronic inflammatory response |
| GO:0032966 | Negative regulation of collagen biosynthetic process |
| GO:2000351 | Regulation of endothelial cell apoptotic process |
| GO:0045667 | Regulation of osteoblast differentiation |
| GO:0071675 | Regulation of mononuclear cell migration |
| GO:0045471 | Response to ethanol |
| GO:0031329 | Regulation of cellular catabolic process |
| GO:1900747 | Negative regulation of vascular endothelial growth factor signaling pathway |
| GO:0050769 | Positive regulation of neurogenesis |
| GO:0001906 | Cell killing |
| GO:0002312 | B cell activation involved in immune response |
| GO:2000317 | Negative regulation of T-helper 17 type immune response |
| GO:0001961 | Positive regulation of cytokine-mediated signaling pathway |
| GO:0051049 | Regulation of transport |
| GO:0031326 | Regulation of cellular biosynthetic process |
| GO:0044089 | Positive regulation of cellular component biogenesis |
| GO:0002674 | Negative regulation of acute inflammatory response |
| GO:0032494 | Response to peptidoglycan |
| GO:0045063 | T-helper 1 cell differentiation |
| GO:0048711 | Positive regulation of astrocyte differentiation |
| GO:0065008 | Regulation of biological quality |
| GO:0001936 | Regulation of endothelial cell proliferation |
| GO:0030858 | Positive regulation of epithelial cell differentiation |
| GO:0050678 | Regulation of epithelial cell proliferation |
| GO:0060284 | Regulation of cell development |
| GO:0050680 | Negative regulation of epithelial cell proliferation |
| GO:0043065 | Positive regulation of apoptotic process |
| GO:0046888 | Negative regulation of hormone secretion |
| GO:0050709 | Negative regulation of protein secretion |
| GO:0048302 | Regulation of isotype switching to IgG isotypes |
| GO:0060221 | Retinal rod cell differentiation |
| GO:0051384 | Response to glucocorticoid |
| GO:0006355 | Regulation of transcription, DNA-templated |
| GO:0062013 | Positive regulation of small molecule metabolic process |
| GO:1905330 | Regulation of morphogenesis of an epithelium |
| GO:0032880 | Regulation of protein localization |
| GO:0150078 | Positive regulation of neuroinflammatory response |
| GO:0010556 | Regulation of macromolecule biosynthetic process |
| GO:0032102 | Negative regulation of response to external stimulus |
| GO:1901224 | Positive regulation of NIK/NF-kappaB signaling |
| GO:0045669 | Positive regulation of osteoblast differentiation |
| GO:1901741 | Positive regulation of myoblast fusion |
| GO:0043524 | Negative regulation of neuron apoptotic process |
| GO:0050796 | Regulation of insulin secretion |
| GO:1901698 | Response to nitrogen compound |
| GO:0010224 | Response to UV-B |
| GO:0051044 | Positive regulation of membrane protein ectodomain proteolysis |
| GO:0051092 | Positive regulation of NF-kappaB transcription factor activity |
| GO:2001267 | Regulation of cysteine-type endopeptidase activity involved in apoptotic signaling pathway |
| GO:0050795 | Regulation of behavior |
| GO:0045623 | Negative regulation of T-helper cell differentiation |
| GO:0051770 | Positive regulation of nitric-oxide synthase biosynthetic process |
| GO:0060252 | Positive regulation of glial cell proliferation |
| GO:2000341 | Regulation of chemokine (C-X-C motif) ligand 2 production |
| GO:0018193 | Peptidyl-amino acid modification |
| GO:0050766 | Positive regulation of phagocytosis |
| GO:0071677 | Positive regulation of mononuclear cell migration |
| GO:0016477 | Cell migration |
| GO:0065009 | Regulation of molecular function |
| GO:0030336 | Negative regulation of cell migration |
| GO:0070498 | Interleukin-1-mediated signaling pathway |
| GO:2000810 | Regulation of bicellular tight junction assembly |
| GO:0034250 | Positive regulation of cellular amide metabolic process |
| GO:0098802 | Plasma membrane signaling receptor complex |
| GO:0005615 | Extracellular space |
| GO:0043235 | Receptor complex |
| GO:0005576 | Extracellular region |
| GO:0009897 | External side of plasma membrane |
| GO:0042022 | Interleukin-12 receptor complex |
| GO:0072536 | Interleukin-23 receptor complex |
| GO:0005887 | Integral component of plasma membrane |
| GO:0009986 | Cell surface |
| GO:0031904 | Endosome lumen |
| GO:0005896 | Interleukin-6 receptor complex |
| GO:0031906 | Late endosome lumen |
| GO:0071944 | Cell periphery |
| GO:0005886 | Plasma membrane |
| GO:0043514 | Interleukin-12 complex |
| GO:0070743 | Interleukin-23 complex |
| GO:0032002 | Interleukin-28 receptor complex |
| GO:0070110 | Ciliary neurotrophic factor receptor complex |
| GO:0030526 | Granulocyte macrophage colony-stimulating factor receptor complex |
| GO:0005125 | Cytokine activity |
| GO:0005126 | Cytokine receptor binding |
| GO:0048018 | Receptor ligand activity |
| GO:0005102 | Signaling receptor binding |
| GO:0004896 | Cytokine receptor activity |
| GO:0005515 | Protein binding |
| GO:0070851 | Growth factor receptor binding |
| GO:0019955 | Cytokine binding |
| GO:0098772 | Molecular function regulator activity |
| GO:0008083 | Growth factor activity |
| GO:0038023 | Signaling receptor activity |
| GO:0030368 | Interleukin-17 receptor activity |
| GO:0004921 | Interleukin-11 receptor activity |
| GO:0005143 | Interleukin-12 receptor binding |
| GO:0019970 | Interleukin-11 binding |
| GO:0019838 | Growth factor binding |
| GO:0005127 | Ciliary neurotrophic factor receptor binding |
| GO:0004920 | Interleukin-10 receptor activity |
| GO:0005139 | Interleukin-7 receptor binding |
| GO:0019972 | Interleukin-12 binding |
| GO:0045509 | interleukin-27 receptor activity |
| GO:0045518 | Interleukin-22 receptor binding |
| GO:0004904 | Interferon receptor activity |
| GO:0005138 | Interleukin-6 receptor binding |

**Table S7:** KEGG enrichment entry.

| **Category** | **Description** |
| --- | --- |
| hsa04060 | Cytokine-cytokine receptor interaction |
| hsa04630 | JAK-STAT signaling pathway |
| hsa05321 | Inflammatory bowel disease |
| hsa04659 | Th17 cell differentiation |
| hsa05200 | Pathways in cancer |
| hsa04640 | Hematopoietic cell lineage |
| hsa04657 | IL-17 signaling pathway |
| hsa04658 | Th1 and Th2 cell differentiation |
| hsa04061 | Viral protein interaction with cytokine and cytokine receptor |
| hsa05152 | Tuberculosis |
| hsa05145 | Toxoplasmosis |
| hsa05323 | Rheumatoid arthritis |
| hsa05140 | Leishmaniasis |
| hsa05164 | Influenza A |
| hsa05144 | Malaria |
| hsa05310 | Asthma |
| hsa04151 | PI3K-Akt signaling pathway |
| hsa05142 | Chagas disease |
| hsa05146 | Amoebiasis |
| hsa05330 | Allograft rejection |
| hsa05143 | African trypanosomiasis |
| hsa05133 | Pertussis |
| hsa05163 | Human cytomegalovirus infection |
| hsa05134 | Legionellosis |
| hsa04620 | Toll-like receptor signaling pathway |
| hsa04066 | HIF-1 signaling pathway |
| hsa05161 | Hepatitis B |
| hsa04664 | Fc epsilon RI signaling pathway |
| hsa05167 | Kaposi sarcoma-associated herpesvirus infection |
| hsa04940 | Type I diabetes mellitus |
| hsa05162 | Measles |
| hsa04217 | Necroptosis |
| hsa04625 | C-type lectin receptor signaling pathway |
| hsa04622 | RIG-I-like receptor signaling pathway |
| hsa05235 | PD-L1 expression and PD-1 checkpoint pathway in cancer |
| hsa04672 | Intestinal immune network for IgA production |
| hsa04933 | AGE-RAGE signaling pathway in diabetic complications |
| hsa05168 | Herpes simplex virus 1 infection |
| hsa04660 | T cell receptor signaling pathway |
| hsa04668 | TNF signaling pathway |
| hsa05130 | Pathogenic Escherichia coli infection |
| hsa04650 | Natural killer cell mediated cytotoxicity |
| hsa05166 | Human T-cell leukemia virus 1 infection |
| hsa04932 | Non-alcoholic fatty liver disease |
| hsa01521 | EGFR tyrosine kinase inhibitor resistance |
| hsa04621 | NOD-like receptor signaling pathway |
| hsa05332 | Graft-versus-host disease |
| hsa04064 | NF-kappa B signaling pathway |
| hsa05169 | Epstein-Barr virus infection |
| hsa04931 | Insulin resistance |
| hsa05132 | Salmonella infection |
| hsa05320 | Autoimmune thyroid disease |
| hsa04380 | Osteoclast differentiation |
| hsa05131 | Shigellosis |
| hsa05135 | Yersinia infection |
| hsa04068 | FoxO signaling pathway |
| hsa04550 | Signaling pathways regulating pluripotency of stem cells |
| hsa05221 | Acute myeloid leukemia |
| hsa04917 | Prolactin signaling pathway |
| hsa04920 | Adipocytokine signaling pathway |
| hsa05160 | Hepatitis C |
| hsa05202 | Transcriptional misregulation in cancer |
| hsa01523 | Antifolate resistance |
| hsa05418 | Fluid shear stress and atherosclerosis |
| hsa04930 | Type II diabetes mellitus |

**Table S8.** Binding energies and RMSD of the ten compounds into the active pocket site of against 3',5"-aminoglycoside phosphotransferase type IIIa (APH(3')-IIIa), (PDB ID: 3TM0).

| **No.** | **Compound** | **S kcal/mol** | **RMSD_refine** |
| --- | --- | --- | --- |
| **1** | **Kaempferol 3-gentiobioside** | -8.1024 | 1.2547 |
| **2** | **Kaempferol 3-sambubioside** | -7.9842 | 1.3698 |
| **3** | **Quercetin 3-rhamnoside** | -7.8726 | 1.4589 |
| **4** | **Procyanidin A2** | -7.5431 | 1.6823 |
| **5** | **Procyanidin A2 3-glucoside** | -7.3256 | 1.7396 |
| **6** | **(2S)-2-hydroxy-3-[[(9Z,12Z)-1-oxo-9,12-octadecadien-1-yl]oxy]propyl-O-β-D-galactopyranoside** | -7.1254 | 1.8532 |
| **7** | **Palmitic acid** | -6.9457 | 1.9214 |
| **8** | **Linoleic acid** | -6.8359 | 2.0156 |
| **9** | **Reference ligand (gentamicin)** | -7.9014 | 1.0289 |
| **10** | **Co-ligand (butirosin A)** | -8.2435 | 0.9543 |
